# Surface tension of nanoparticle dispersions unravelled by size-dependent non-occupied sites free energy versus adsorption kinetics

**DOI:** 10.1038/s41526-022-00234-3

**Published:** 2022-11-03

**Authors:** Hatim Machrafi

**Affiliations:** 1 Université de Liège, Institut de Physique, Liège, 4000 Belgium; 2 Université libre de Bruxelles, Physical Chemistry Group, Bruxelles, 1050 Belgium; 3 Sorbonne Université, UFR Physique, Paris, 75005 France

**Keywords:** Colloids, Surfaces, interfaces and thin films, Fluid dynamics

## Abstract

The surface tension of dispersions presents many types of behaviours. Although some models, based on classical surface thermodynamics, allow partial interpretation, fundamental understanding is still lacking. This work develops a single analytical physics-based formulation experimentally validated for the surface tension of various pure nanoparticle dispersions, explaining the underlying mechanisms. Against common belief, surface tension increase of dispersions appears not to occur at low but rather at intermediate surface coverage, owed by the relatively large size of nanoparticles with respect to the fluid molecules. Surprisingly, the closed-form model shows that the main responsible mechanism for the various surface tension behaviours is not the surface chemical potential of adsorbed nanoparticles, but rather that of non-occupied sites, triggered and delicately controlled by the nanoparticles ‘at a distance’, introducing the concept of the ‘non-occupancy’ effect. The model finally invites reconsidering surface thermodynamics of dispersions and provides for criteria that allow in a succinct manner to quantitatively classify the various surface tension behaviours.

## Introduction

Fluid dynamics of complex fluids represent a field of study that concerns a series of energetic, medical and industrial engineering applications. Since these applications concern, in many cases, fluids wherein dispersions are used for material printing or separation processes at the surface level, it is important to control the behaviour of surface-related mechanisms^1^. Stability requirements during the dispersion processing and the printing process depend on the physical properties, such as the viscosity, and its deposition quality depends on controlling the fluid dynamics of the deposited fluids and the underlying mechanisms^2,3^. Moreover, the wettability is a relevant physical property for processes where droplet impingement, thin film flows, microfluidics, surface speciation and heat transfer are implied^4,5^.

In order to focus on surface-related mechanisms of complex fluid dynamics, microgravity experiments are useful, cancelling thereby the interference of buoyancy. A sounding rocket experiment took place under the framework of the Advanced Research on Liquid Evaporation in Space (ARLES) experiment supported by the European Space Agency (ESA). ARLES was part of the payload in a SubOrbital Express rocket (MASER 14) and aims at studying the evaporation of pure and complex sessile droplets. It also serves as a preparation of an experiment to be performed in the near future at the European Drawer Rack 2 on board the International Space Station. The evaporation of the complex droplets resulted into pattern depositions of nanoparticles, interesting for future printing applications. These experiments allowed studying the depositions, but not how fluid dynamics, surface effects and particle–fluid interactions controlled those depositions. Another sounding rocket experiment is planned to be performed in the near future, where one of the focuses will be to monitor the fluid dynamics of the complex droplets. In order to prepare the flight scenario, a numerical model has been developed. The condition expressing the tangential stress balance, including surface-tension-induced convection, i.e. Marangoni convection, at the interface is given by

1 $$- (\sigma _{{{\mathrm{g}}}} \cdot {{{\mathbf{n}}}}) + (\sigma _{{{\mathrm{l}}}} \cdot {{{\mathbf{n}}}}) + \gamma (\nabla \cdot {{{\mathbf{n}}}})_{\Sigma}{{{\mathbf{n}}}} - \gamma _T\nabla _{\Sigma}T - \gamma _\varphi \nabla _{\Sigma}\varphi = 0$$

where ***σ***_g_ and ***σ***_l_ are the stress tensors at the interface on the gas and liquid sides, respectively, **n** the normal vector on the interface, *γ* the surface tension, (∇∙**n**)_Σ_ stands for the curvature of the interface, ∇_Σ_ = ∇ − **nn** ∙ ∇ for the surface gradient, *T* and *φ* stand for the temperature and nanoparticle volume fraction at the interface, respectively, whereas *γ*_*T*_ defines the surface tension derivative with respect to the temperature and *γ*_*φ*_ the surface tension derivative with respect to the volume fraction. Generally, *γ*_*T*_ is readily available and reasonably approximated to be constant. However, the behaviour of *γ*_*φ*_ is not so clear. Since at microgravity, convection responsible for fluid patterns is of the surface-tension type, it is crucial to not only have a physics-based analytical expression for the surface tension of nanoparticle-laden fluids but also to understand the underlying mechanisms that govern the surface tension of such complex fluids.

It appears from several experimental studies that apparently contradictory tendencies of the surface tension as a function of nanoparticle content are observed: the surface tension is observed to increase, decrease or pass through a minimum as a function of the nanoparticle content in the dispersions^6–15^. Even for the same nanoparticle, e.g. SiO_2_, a constant and increasing trend is observed^9,13^, while for Al_2_O_3_ both decreasing and increasing trends, in two separate cases, are observed^7^. Due to the diversity of nanodispersions, there is no universal relation yet that could comprehend and clarify quantitatively such observed trends. Fitted correlations or empiric relations may give good comparison to experimental values, but are specific to experiment conditions, do not explain physically why certain surface tension phenomena occur and lack often generality^7,10^. One may find some explanations, but mostly based on a qualitative assessment of the Gibbs free energy or on mere intuition. Some works have explained surface tension behaviour by an energy variation upon nanoparticle transfer to the interface^16^, attractive van der Waals or attractive capillary forces^6,17^ or even by analogy with electrolyte solutions^18^. Interestingly, it is just the presence of nanoparticles at the surface that is given as reason for surface tension increase in ref. ^11^, while the nanoparticle adsorption is suggested to cause a decrease in the surface tension elsewhere^17^. Others explain surface tension decrease by a high ionic strength of the base fluid countering the otherwise repulsive force between the nanoparticles and the liquid–gas interface^18^. This, however, does not explain the decrease of surface tension of nanoparticle-laden fluids with low ionic strength, such as nanoparticle dispersions in distilled water^19^ nor for surface tension minima. The initial decrease of the surface tension is suggested to be due to the large spacing between the nanoparticles, favouring electrostatic forces between the nanoparticles^6^ or to initial adsorption of nanoparticles at the liquid–gas interface^17^.

The apparently contradictory explanations for surface tension behaviour are often intuitively provided and many existing models, useful as they might be, only predict part of the tendencies, which is a consequence of universal underlying mechanisms still remaining unelucidated. This work develops an analytical model proposing a new insight in surface thermodynamics, surface energy and, more particularly, in the interaction between the dispersed phase and the liquid–gas interface. The model mainly aims at elucidating the underlying mechanisms of the surface tension of nanoparticle dispersions, both correctly predicting and explaining thereby the different experimentally observed tendencies. We start by formulating the framework within which the liquid-gas interface is defined. This will also allow introducing definitions of nanoparticle (excess) surface concentrations based on geometrical and size considerations. The nanoparticles are modelled as being incompressible, non-stretchable and the only material that can be adsorbed on the liquid–gas interface. Then, an analytical expression for the surface tension of nanoparticles will be calculated and compared to several experimental data of different nanoparticle dispersions. The model will be used to explain the different observed phenomena. Finally, it will be shown that two parameters can predict the type of surface tension behaviour for all the considered material systems.

## Methods

### Representation of an interfacial layer on a dividing surface

The surface energy of a nanoparticle dispersion can commonly be described by Gibbs adsorption isotherm ${{{\mathrm{d}}}}\gamma = - {{{\mathrm{{\Gamma}}}}}_{{{\mathrm{p}}}}{{{\mathrm{d}}}}\eta _{{{\mathrm{p}}}}^{{{\mathrm{{\Sigma}}}}} - {{{\mathrm{{\Gamma}}}}}_{{{\mathrm{f}}}}{{{\mathrm{d}}}}\eta _{{{\mathrm{f}}}}^{{{\mathrm{{\Sigma}}}}}$, an exact differential. Here, $\eta _{{{\mathrm{p}}}}^{{{\mathrm{{\Sigma}}}}}$ and $\eta _f^{{{\mathrm{{\Sigma}}}}}$ are the surface chemical potentials induced by nanoparticle and fluid surface coverage, respectively, whereas *Γ*_p_ and *Γ*_f_ stand, respectively, for the excess surface concentrations of the nanoparticles and the base fluid. Choosing the Gibbs dividing surface to be there where the excess surface concentration of the liquid equals zero, we set *Γ*_f_ ≡ 0. This leaves us with ${{{\mathrm{d}}}}\gamma = - {\Gamma}_{{{\mathrm{p}}}}{{{\mathrm{d}}}}\eta _{{{\mathrm{p}}}}^{{{\mathrm{{\Sigma}}}}}$. We will start discussing the excess surface concentration first. This inherently entails the definition of a framework that explains how to deal with the representation of a three-dimensional interfacial layer, whilst Gibbs adsorption isotherm imposes to work with a two-dimensional one, i.e. the Gibbs dividing surface. As excess surface concentrations of nanoparticles *Γ*_p_ are not widely documented, such a framework will allow us to propose analytical expressions for *Γ*_p_. The excess surface concentration is composed out of a surface equivalent of the bulk concentration, discussed later, and an actual surface concentration. Let us begin with the latter. The surface concentration of the nanoparticles *Γ*_p_^Σ^ is defined by the surface coverage *θ*_p_ times the maximum surface concentration ${{{\mathrm{{\Gamma}}}}}_{{{{\mathrm{p}}}},{{{\mathrm{max}}}}}^{{{\mathrm{{\Sigma}}}}}$:

2 $${{{\mathrm{{\Gamma}}}}}_{{{\mathrm{p}}}}^{\Sigma} = \theta _{{{\mathrm{p}}}}{{{\mathrm{{\Gamma}}}}}_{{{{\mathrm{p}}}},{{{\mathrm{max}}}}}^{\Sigma},$$

the surface coverage *θ*_p_ stems from principles concerning thermodynamic equilibrium and adsorption kinetics and it is more appropriate to discuss it later in a proper context. For now, we will focus on the framework of the dividing surface and how the surface concentration is represented within its context.

**Fig. 1:**
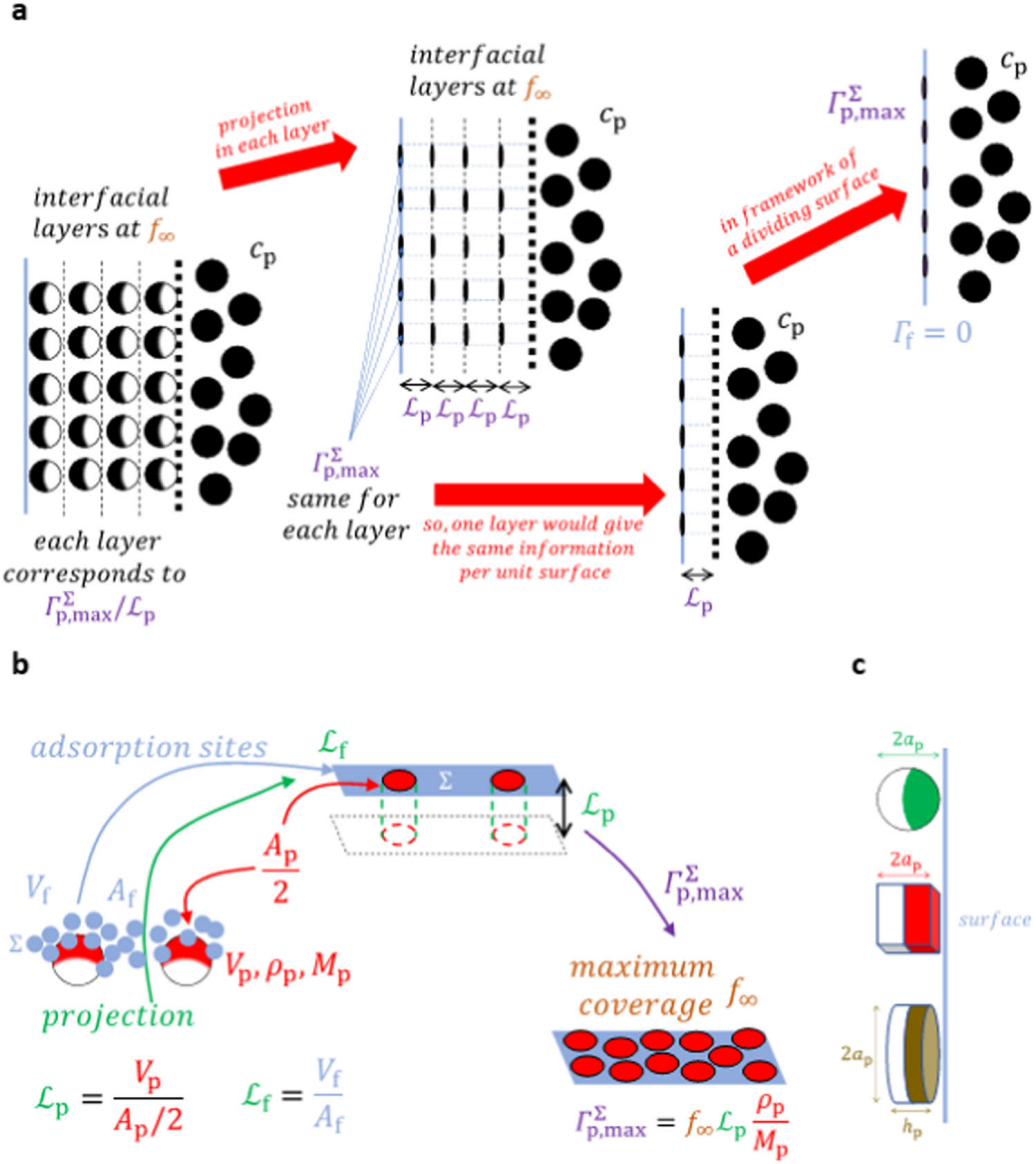
The projection of nanoparticles on the dividing surface. **a** Principle of projecting nanoparticles on dividing surface at maximum coverage. Note that at a coverage below the maximum one, the principle is the same. **b** Projection of nanoparticles (with volume *V*_P_ and surface *A*_P_) on a two-dimensional surface. The projected circles are oval because they are drawn in perspective, but they should be seen as circular for the spherical and disk nanoparticles, and as a square for the cubic nanoparticles. The fluid molecules (with volume *V*_P_ and surface *A*_P_) at the surface Σ, on which a nanoparticle adsorbs, also participate to the adsorption and are illustrated as fluid molecules that become projected (on the dividing surface) as two-dimensional adsorption sites, depicted for simplicity as a flat plane Σ at exactly the dividing surface. **c** Illustration of the surfaces that come into play in the volume-to-surface ratio for the spherical, cubical and disk nanoparticles. The images are not in scale.

In Eq. (2), ${\Gamma}_{{{{\mathrm{p}}}},{{{\mathrm{max}}}}}^{{{\mathrm{{\Sigma}}}}}$ is the maximum surface concentration of the nanoparticles, assumed to be determined by the principle of maximum stacking via a maximum coverage fraction *f*_∞_^20–22^. Other geometrical considerations of particle adsorption have been treated in refs. ^21,22^, but we only need here their results for maximum coverage. In the presence of nanoparticles, the liquid–gas layer can be defined as the layer where the nanoparticles go gradually from a bulk concentration *c*_p_ to a purely surface concentration *Γ*_p_^Σ^. The surface concentration is then usually obtained by integrating the concentration profile over the thickness of that layer. It has therefore, generally, a thickness that is larger than the size of the nanoparticles, a thickness that is defined by the difference between the dividing surface and an imaginary parallel surface, beyond which the bulk concentration is attained. The degree of strength of the interaction energy between the fluid molecules and the nanoparticles in that layer will determine the amount of nanoparticles that are ‘trapped’, i.e. adsorbed, or allowed to disperse in the bulk. Each nanoparticle that adsorbs will push away fluid molecules. In analogy to the bulk, according to the lattice model, (where each lattice fits a liquid molecule), we can define that the surface area that is occupied by a fluid molecule harbours a possible adsorption site. As such, the adsorption sites are *geometrically* equivalent to the fluid molecules in the interfacial layer. In order to represent this framework in a manner that fits Gibbs’ isotherm, we have defined a dividing surface. Speaking of the maximum surface concentration, this also necessitates to project the real maximum concentration in the interfacial layer onto the dividing surface (that has zero thickness). This projection method is depicted in Fig. 1a, while Fig. 1b focusses on the projection of an adsorbing nanoparticle with the corresponding parameters that will be used in the model. Figure 1c shows the surface that is deemed to participate to the adsorption. In fact, upon adsorption, it can be imagined that not the whole surface of the nanoparticles participates in the process. It is quite difficult to assess the portion of surface that participates to this process and not much is known about this. In this work, we will heuristically assume that only half of a nanoparticle’s surface, i.e. the half that faces the dividing surface, participates in its adsorption. The reason for this will be discussed later. We will call this the participating surface. Later in ‘*Three counter-intuitive effects of K*_*p*_ *on surface tension*’, a verification will be discussed to show that such an assumption appears to be quite reasonable.

Let us, at maximum coverage, order the nanoparticles into several layers that are parallel to the dividing surface. As we are in maximum coverage, *each layer* contains the same amount of nanoparticles. Let us then project, *in each such layer*, the participating surface of the nanoparticles (as if a nanoparticle was a balloon that is cut in half, of which one half is spread over the surface) on the dividing surface (for the first layer at the dividing surface) and on subsequent imaginary layers parallel to the dividing surface. This gives in each layer the same maximum surface concentration per unit surface, so that considering only the layer adjacent to the dividing surface is sufficient to determine the maximum surface concentration *per unit surface*, as Fig. 1a shows.

This will result in a molar concentration of nanoparticles per unit surface on the dividing surface that would be equivalent to the corresponding real molar concentration in a realistic adsorption layer, through scaling by a certain defined characteristic length, so-defined as ${{{\mathcal{L}}}}_{{{\mathrm{p}}}}$. As Fig. 1b shows, the projected maximum surface concentration per unit surface times the characteristic length gives the same volume as that of the nanoparticle. It follows then that that characteristic length must be a volume-to-surface ratio.

We define this volume-to-surface ratio by the total volume divided by half of the total surface (the participating surface, as is shown in Fig. 1c). The fluid particles that surround this participating surface of a nanoparticle in a real interfacial layer are fully projected on the dividing surface as they represent geometrically the adsorption sites on that surface (see the schematic representation in Fig. 1b). The characteristic length of these fluid particles, or adsorption sites, is calculated by a standard volume-to-surface ratio.

The projected surfaces depend on the size of the nanoparticles and the fluid molecules (geometrically equivalent to that of the adsorption sites). This means that the difference in sizes between the nanoparticles and the adsorption sites can be expected to have a great impact on the adsorption process. Each adsorbed nanoparticle will induce a change in the possible entropic configurations of a great number of adsorption sites. As these sites are not occupied, yet have a large influence on the entropy, they are named as non-occupied sites, because their non-occupancy matters entropically. We can say that the total area of these non-occupied sites (denoted by subscript *NO*) per total surface area is given by

3 $$\varsigma _{{{{\mathrm{NO}}}}}m_{{{{\mathrm{NO}}}}}^{{{\mathrm{{\Sigma}}}}} = \varsigma _{{{\mathrm{f}}}}m_{{{\mathrm{f}}}}^{{{\mathrm{{\Sigma}}}}} - \varsigma _{{{\mathrm{p}}}}m_{{{\mathrm{p}}}}^{{{\mathrm{{\Sigma}}}}}$$

where $m_{{{\mathrm{i}}}}^{{{\mathrm{{\Sigma}}}}}$ and *ς*_i_ are, respectively, the number of particles of a constituent per unit interface surface and the specific surface area per particle of that constituent, whilst the superscript Σ indicates that it concerns a surface property. The specific surface area of a constituent is given by $\varsigma _{{{\mathrm{i}}}} \equiv \frac{{M_{{{\mathrm{i}}}}}}{{\rho _{{{\mathrm{i}}}}{{{\mathcal{L}}}}_{{{\mathrm{i}}}}N_{{{\mathrm{A}}}}}}$, with *i* = *NO*, *f*, *p*, standing for non-occupied sites, the fluid and nanoparticles, respectively. Equivalently, the specific surface area of a constituent may conveniently also be given per *mole*, i.e. $\varsigma _{{{\mathrm{i}}}} \equiv \frac{{M_{{{\mathrm{i}}}}}}{{\rho _{{{\mathrm{i}}}}{{{\mathcal{L}}}}_{{{\mathrm{i}}}}}}$. In that case, $m_{{{\mathrm{i}}}}^{{{\mathrm{{\Sigma}}}}}$ would simply be the number of *mole* of a constituent per unit surface via scaling by *N*_A_ and Eq. (3) remains valid. Expressions for the characteristic length ${{{\mathcal{L}}}}_{{{\mathrm{i}}}}$ will be developed later (with ${{{\mathcal{L}}}}_{{{{\mathrm{NO}}}}} = {{{\mathcal{L}}}}_{{{\mathrm{f}}}}$ as geometrically the adsorption sites are equivalent to the fluid molecules). It is important to notice here that *ς*_i_ introduces a size effect, i.e. the number densities depend on the size of the nanoparticles and the surface fluid particles.

We have now defined the framework for calculating the surface concentrations and may proceed with proposing a formula that allows calculating these concentrations. The maximum surface concentration is given by the maximum number of nanoparticle moles, $\frac{{m_{{{{\mathrm{p}}}},{{{\mathrm{max}}}}}}}{{N_{{{\mathrm{A}}}}}}$, divided by a unit surface, i.e. ${\Gamma}_{{{{\mathrm{p}}}},{{{\mathrm{max}}}}}^{{{\mathrm{{\Sigma}}}}} = \frac{1}{{N_{{{\mathrm{A}}}}}}\frac{{m_{{{{\mathrm{p}}}},{{{\mathrm{max}}}}}}}{{A_{{{\mathrm{t}}}}}}$, where *A*_t_ is a total (arbitrary portion of unit) surface. This can conveniently be rewritten as ${\Gamma}_{{{{\mathrm{p}}}},{{{\mathrm{max}}}}}^{{{\mathrm{{\Sigma}}}}} = \frac{{V_{{{\mathrm{p}}}}}}{{A_{{{\mathrm{p}}}}/2}}\frac{{m_{{{{\mathrm{p}}}},{{{\mathrm{max}}}}}A_{{{\mathrm{p}}}}/2}}{{A_{{{\mathrm{t}}}}}}\frac{1}{{N_{{{\mathrm{A}}}}V_{{{\mathrm{p}}}}}}$, where *A*_p_ and *V*_p_ are the surface and volume per nanoparticle, respectively. It can be noted that $\frac{{V_{{{\mathrm{p}}}}}}{{A_{{{\mathrm{p}}}}/2}}$ is nothing else than two times the nanoparticle’s volume to surface ratio, defined as ${{{\mathcal{L}}}}_{{{\mathrm{p}}}}$ (see Fig. 1). Moreover, $\frac{1}{{N_{{{\mathrm{A}}}}V_{{{\mathrm{p}}}}}}$ is the mole of nanoparticles per unit volume, also given by $\frac{{\rho _{{{\mathrm{p}}}}}}{{M_{{{\mathrm{p}}}}}}$, where *ρ*_p_ and *M*_p_ are the nanoparticle’s density and molar mass, respectively. Also $\frac{{m_{{{{\mathrm{p,max}}}}}A_{{{\mathrm{p}}}}/2}}{{A_{{{\mathrm{t}}}}}}$ is simply the maximum geometric coverage fraction *f*_∞_, being *f*_∞_ ≈ 0.547 for spherical non-overlapping hard particles on a two-dimensional surface^21,22^. This value is obtained by considering random packing of spheres after their projection on the interface. It is therefore not the same as the random packing of circles as the latter would neglect the purpose of the projection method, where it is sought to obtain expressions on a 2D surface whilst preserving the information from realistic 3D interfaces as Fig. 1 shows. For cubic nanoparticles, it is reasonable to expect that the maximum coverage will be close to unity, to that we take *f*_∞_ ≈ 1 for cubic nanoparticles^21,22^. For disk-like nanoparticles, of which the circular part faces the dividing surface, we take a maximum coverage that corresponds to maximum standard hexagonal stacking of circles on a surface, i.e. $f_\infty = \frac{{\pi \sqrt 3 }}{6} \approx 0.907$ for disk nanoparticles. This leads to

4 $${\Gamma}_{p,\max }^{\Sigma} = f_\infty {{{\mathcal{L}}}}_p\frac{{\rho _p}}{{M_p}}$$

It should be noted that when nanoparticles are coated or surface treated, the maximum coverage might be less due to possible repulsive forces or more if the nanoparticles have a soft compressible coating with interparticle attractive forces. Note that similar expressions have been proposed in refs. ^20,23^. Nanoparticles may come in various shapes, the main ones kibeing often of spherical, cubical or disk shape. We will, for the demonstration, limit ourselves to such undeformable shapes. The volume and participating surface, i.e. $\left( {V_{{{\mathrm{p}}}},\;\frac{{A_{{{\mathrm{p}}}}}}{2}} \right)$, as defined in Fig. 1c, would then be $\left( {\frac{{4\pi }}{3}a_{{{\mathrm{p}}}}^3,\;2\pi a_{{{\mathrm{p}}}}^2} \right)$, $\left( {8a_{{{\mathrm{p}}}}^3,\;12a_{{{\mathrm{p}}}}^2} \right)$ and $\left( {\pi a_{{{\mathrm{p}}}}^2h_{{{\mathrm{p}}}},\;\pi a_{{{\mathrm{p}}}}^2 + \pi a_{{{\mathrm{p}}}}h_{{{\mathrm{p}}}}} \right)$ for a spherical, cubical and disk nanoparticle, respectively. The volume-to-surface ratio ${{{\mathcal{L}}}}_{{{\mathrm{p}}}}$ can then be calculated. For a spherical nanoparticle, ${{{\mathcal{L}}}}_{{{\mathrm{p}}}} = \frac{{2a_{{{\mathrm{p}}}}}}{3}$ (with radius *a*_p_), for a square-like nanoparticle, ${{{\mathcal{L}}}}_{{{\mathrm{p}}}} = \frac{{2a_{{{\mathrm{p}}}}}}{3}$ (with 2*a*_p_ the side of the cube) and for a disk-like nanoparticle, ${{{\mathcal{L}}}}_{{{\mathrm{p}}}} = \frac{{a_{{{\mathrm{p}}}}h_{{{\mathrm{p}}}}}}{{a_{{{\mathrm{p}}}}\, + \,h_{{{\mathrm{p}}}}}}$ (with radius *a*_p_ and thickness *h*_p_). Note that it is not straightforward to define a molar mass of a nanoparticle, since it is not a molecule nor an atom. We will approximate the molar mass of a nanoparticle, in analogy with that of a polymer constituted by many monomers, as an ensemble of atoms or molecules chemically connected to one another. The molar mass of a nanoparticle equals then $M_{{{\mathrm{p}}}} = M_{{{{\mathrm{p}}}}^\prime }f_{{{\mathrm{p}}}}\frac{{V_{{{\mathrm{p}}}}}}{{V_{{{{\mathrm{p}}}}^\prime }}}$, where *f*_p_ represents the maximum stacking factor of spheres in a three-dimensional setting, assumed here to be $f_{{{\mathrm{p}}}} = \frac{{\uppi }}{{3\sqrt 2 }}$ (that of an fcc or hcp structure), *M*_p′_ the molar mass of one atom or molecule, *V*_p_ the volume of one nanoparticle and $V_{{{{\mathrm{p}}}}^\prime } = \frac{{4{\uppi}}}{3}\ell _{{{\mathrm{p}}}}^3$ the volume of one atom or molecule, assumed to be of spherical form, with $\ell _{{{\mathrm{p}}}}$ the radius of that atom or molecule $\ell _{{{\mathrm{p}}}} = \root {3} \of {{\frac{{M_{{{{\mathrm{p}}}}^\prime }}}{{\rho _{{{\mathrm{p}}}}N_{{{\mathrm{A}}}}}}\frac{3}{{4{\uppi}}}}}$. The effective radius of a fluid molecule, assuming sphericity, can be calculated in the same manner as $\ell _{{{\mathrm{f}}}} = \root {3} \of {{\frac{{M_{{{\mathrm{f}}}}}}{{\rho _{{{\mathrm{f}}}}N_{{{\mathrm{A}}}}}}\frac{3}{{4{\uppi}}}}}$, where *ρ*_f_ and *M*_f_ are the fluid’s density and molar mass, respectively. With $V_{{{\mathrm{f}}}} = \frac{{4\pi }}{3}\ell _{{{\mathrm{f}}}}^3$ and $A_{{{\mathrm{f}}}} = 4\pi \ell _{{{\mathrm{f}}}}^2$, the volume-to-surface ratio for a fluid particle is given by ${{{\mathcal{L}}}}_{{{\mathrm{f}}}} = \frac{{\ell _{{{\mathrm{f}}}}}}{3}$. With these definitions, the geometric definition of the maximum surface concentration *Γ*_p_^Σ^ can be calculated from material properties and size values.

**Fig. 2:**
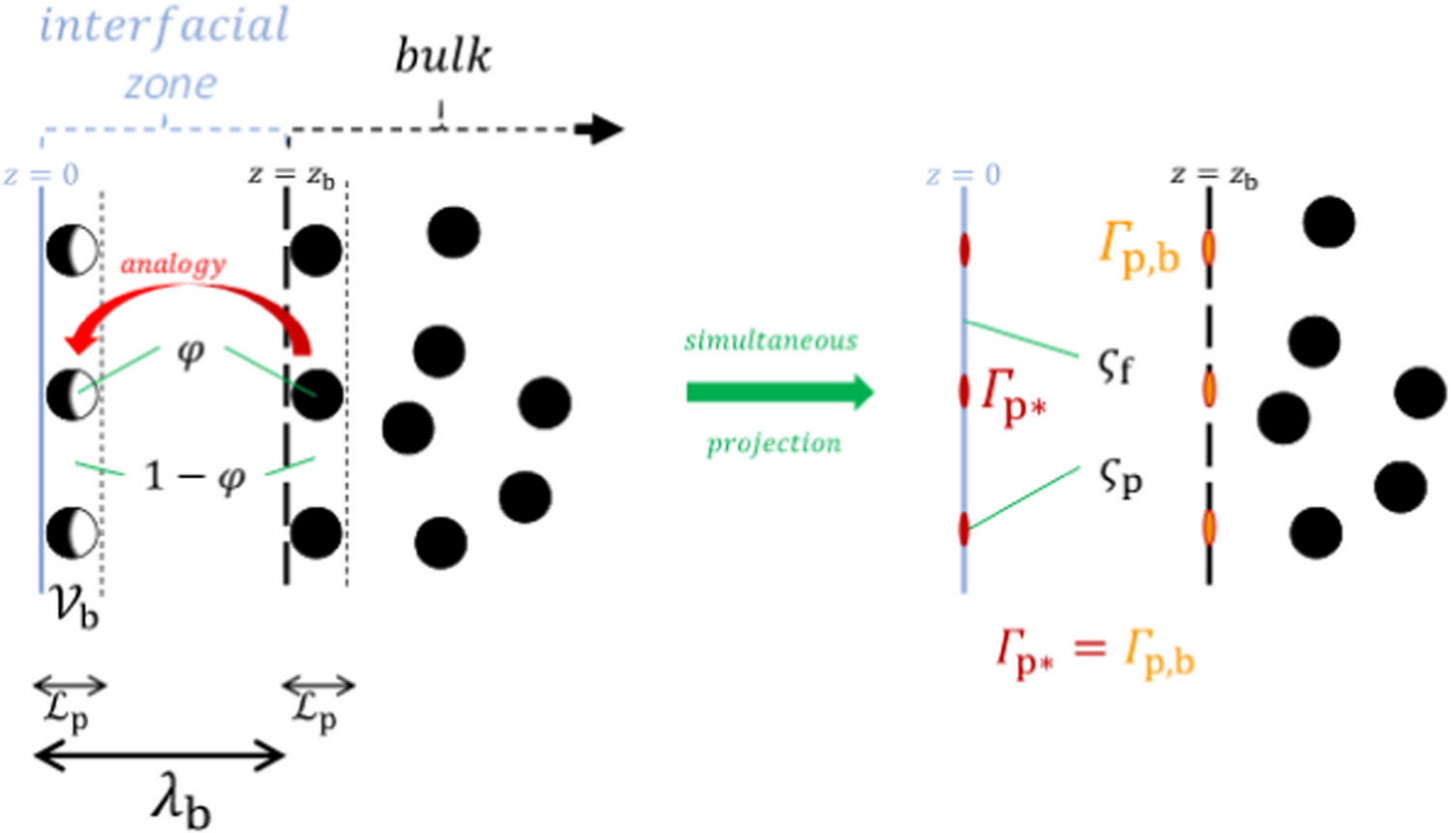
Projection of bulk concentration. Analogy of an imaginary projection of the nanoparticles at an imaginary surface at *z* = *z*_b_ with respect to the real projection on the dividing surface at *z* = 0. The two resulting molar concentrations per unit surface should be equal in the definition of the surface excess concentration.

The difference between the excess surface concentration *Γ*_p_ and the surface concentration *Γ*_p_^Σ^ is usually defined as

5 $${{{\mathrm{{\Gamma}}}}}_{{{\mathrm{p}}}} - {{{\mathrm{{\Gamma}}}}}_{{{\mathrm{p}}}}^{{{\mathrm{{\Sigma}}}}} \equiv - {\int_{z_0}^{z_{{{\mathrm{b}}}}}} {c_{{{\mathrm{p}}}}{{{\mathrm{d}}}}z = - c_{{{\mathrm{p}}}}\lambda _{{{\mathrm{b}}}}}$$

where *c*_p_ is the nanoparticle concentration in the bulk layer, and *λ*_b_ = *z*_b_ − *z*_0_, with *z*_0_ the position at which *Γ*_f_ = 0 and *z*_b_ at which we can consider conceptually to have a bulk concentration. In order to determine *λ*_b_, let us make a preliminary remark. The maximum surface concentration has been obtained by projecting a layer of adsorbed nanoparticles on a two-dimensional surface at the dividing surface, which we defined as *Γ*_f_ = 0. When doing this, it has been explained that the characteristic length of nanoparticle projection equals ${{{\mathcal{L}}}}_{{{\mathrm{p}}}}$. For consistency, we should do the same here. In the definition of the excess surface concentration, the term $\left( { - {\int}_{z_0}^{z_{{{\mathrm{b}}}}} {c_{{{\mathrm{p}}}}{{{\mathrm{d}}}}z} } \right)$ denotes a deduction from the surface concentration of an imaginary extrapolation of the bulk concentration, integrated over the interfacial thickness *λ*_b_. So, it should rather be seen as an imaginary surface-equivalent of the bulk concentration, ${\Gamma}_{{{{\mathrm{p}}}},{{{\mathrm{b}}}}} \equiv {\int}_{z_0}^{z_{{{\mathrm{b}}}}} {c_{{{\mathrm{p}}}}{{{\mathrm{d}}}}z = c_{{{\mathrm{p}}}}\lambda _{{{\mathrm{b}}}}}$, defined at an imaginary surface at *z* = *z*_b_. This also means that it is analogous to the projection of the bulk nanoparticle concentration on the dividing surface, named here *Γ*_p*_, so that *Γ*_p*_ ≡ *Γ*_p,b_. It remains to find *Γ*_p*_. Imagine at the dividing surface *Γ*_f_ = 0 a slab ${{{\mathcal{V}}}}_{{{\mathrm{p}}}}$ of thickness ${{{\mathcal{L}}}}_{{{\mathrm{p}}}}$, of which the contents are projected on that surface. If the projected nanoparticle surface concentration is given by *Γ*_p*_, then the corresponding nanoparticle concentration in ${{{\mathcal{V}}}}_{{{\mathrm{p}}}}$ would be $\frac{{{\Gamma}_{{{{\mathrm{p}}}} \ast }}}{{{{{\mathcal{L}}}}_{{{\mathrm{p}}}}}}$ as defined by the projection procedure in Fig. 1. If the projected specific surface area per mole of nanoparticles is given by *ς*_p_, then the corresponding volume per mole of nanoparticles in ${{{\mathcal{V}}}}_{{{\mathrm{p}}}}$ would be $\varsigma _{{{\mathrm{p}}}}{{{\mathcal{L}}}}_{{{\mathrm{p}}}}$. The same could be done for the fluid particles, so that the volume fraction *φ* in that slab would be described by $\frac{\varphi }{{1 - \varphi }} = \frac{{\varsigma _{{{\mathrm{p}}}}{\Gamma}_{{{{\mathrm{p}}}} \ast }}}{{\varsigma _{{{\mathrm{f}}}}{\Gamma}_{{{{\mathrm{f}}}} \ast }}}$. Within the slab ${{{\mathcal{V}}}}_{{{\mathrm{p}}}}$, the projected surface concentration for the fluid particles *Γ*_f*_ would simply be equal to the bulk concentration *c*_f_ times the thickness of ${{{\mathcal{V}}}}_{{{\mathrm{p}}}}$, i.e. ${\Gamma}_{{{{\mathrm{f}}}} \ast } = c_{{{\mathrm{f}}}}{{{\mathcal{L}}}}_{{{\mathrm{p}}}}$. We then have ${\Gamma}_{{{{\mathrm{p}}}} \ast } = c_{{{\mathrm{f}}}}{{{\mathcal{L}}}}_{{{\mathrm{p}}}}\frac{{\varsigma _{{{\mathrm{f}}}}}}{{\varsigma _{{{\mathrm{p}}}}}}\frac{\varphi }{{1 - \varphi }}$. Note that $c_{{{\mathrm{f}}}} = \left( {1 - \varphi } \right)\frac{{\rho _{{{\mathrm{f}}}}}}{{M_{{{\mathrm{f}}}}}}$. Filling in the definitions of *ς*_p_ and *ς*_f_ leads to ${\Gamma}_{{{{\mathrm{p}}}} \ast } = \frac{{\rho _{{{\mathrm{f}}}}}}{{M_{{{\mathrm{f}}}}}}{{{\mathcal{L}}}}_{{{\mathrm{p}}}}\frac{{M_{{{\mathrm{f}}}}}}{{\rho _{{{\mathrm{f}}}}{{{\mathcal{L}}}}_{{{\mathrm{f}}}}}}\left( {\frac{{M_{{{\mathrm{p}}}}}}{{\rho _{{{\mathrm{p}}}}{{{\mathcal{L}}}}_{{{\mathrm{p}}}}}}} \right)^{ - 1}\varphi = \frac{{\rho _{{{\mathrm{p}}}}}}{{M_{{{\mathrm{p}}}}}}\frac{{{{{\mathcal{L}}}}_{{{\mathrm{p}}}}^2}}{{{{{\mathcal{L}}}}_{{{\mathrm{f}}}}}}\varphi$. Figure 2 illustrates the analogy that we have discussed here.

As $c_{{{\mathrm{p}}}} = \varphi \frac{{\rho _{{{\mathrm{p}}}}}}{{M_{{{\mathrm{p}}}}}}$, it follows that $\lambda _{{{\mathrm{b}}}} = \frac{{{{{\mathcal{L}}}}_{{{\mathrm{p}}}}^2}}{{{{{\mathcal{L}}}}_{{{\mathrm{f}}}}}}$. Filling this in (5) gives, with Eq. (2), for the excess surface concentration

6 $${{{\mathrm{{\Gamma}}}}}_{{{\mathrm{p}}}} \equiv \theta _{{{\mathrm{p}}}}{{{\mathrm{{\Gamma}}}}}_{{{{\mathrm{p}}}},{{{\mathrm{max}}}}}^{{{\mathrm{{\Sigma}}}}} - \varphi {{{\mathrm{{\Gamma}}}}}_{{{\mathrm{b}}}}^{{{\mathrm{{\Sigma}}}}} = \left( {\theta _{{{\mathrm{p}}}}K_{{{\mathrm{{\Sigma}}}}} - \varphi } \right){{{\mathrm{{\Gamma}}}}}_{{{\mathrm{b}}}}^{{{\mathrm{{\Sigma}}}}}$$

with

7 $${{{\mathrm{{\Gamma}}}}}_{{{\mathrm{b}}}}^{{{\mathrm{{\Sigma}}}}} = \frac{{c_{{{\mathrm{p}}}}}}{\varphi }\lambda _{{{\mathrm{b}}}} = \frac{{\rho _{{{\mathrm{p}}}}}}{{M_{{{\mathrm{p}}}}}}\frac{{{{{\mathcal{L}}}}_{{{\mathrm{p}}}}^2}}{{{{{\mathcal{L}}}}_{{{\mathrm{f}}}}}}$$

8 $$K_{{{\mathrm{{\Sigma}}}}} = \frac{{{\Gamma}_{{{{\mathrm{p}}}},{{{\mathrm{max}}}}}^{{{\mathrm{{\Sigma}}}}}}}{{{\Gamma}_{{{\mathrm{b}}}}^{{{\mathrm{{\Sigma}}}}}}}$$

where ${\Gamma}_{{{{\mathrm{p}}}},{{{\mathrm{max}}}}}^{{{\mathrm{{\Sigma}}}}}$ is given by Eq. (4) and *φΓ*_b_^Σ^ is the surface equivalent of the bulk concentration and *K*_Σ_ a constant that measures the potential of the nanoparticles to rather adsorb at the interface than stay dispersed in the bulk. As will be seen later, *K*_Σ_ is a function of nanoparticle size, maximum packing and fluid molecule size. The surface coverage *θ*_p_ in Eq. (6) will be treated in the context of surface kinetics, but we will first deduce the surface chemical potentials and surface adsorption.

### Surface chemical potential

If we have $m_{{{\mathrm{f}}}}^{{{\mathrm{{\Sigma}}}}}$ number densities of adsorption sites, containing $m_{{{{\mathrm{NO}}}}}^{{{\mathrm{{\Sigma}}}}}$ number densities of non-occupied sites and $m_{{{\mathrm{p}}}}^{{{\mathrm{{\Sigma}}}}}$ number densities of adsorbed nanoparticles, the total number of microstates would equal $W = \frac{{\left( {m_{{{{\mathrm{NO}}}}}^{{{\mathrm{{\Sigma}}}}} + m_{{{\mathrm{p}}}}^{{{\mathrm{{\Sigma}}}}}} \right)!}}{{m_{{{\mathrm{p}}}}^{{{\mathrm{{\Sigma}}}}}!m_{{{{\mathrm{NO}}}}}^{{{\mathrm{{\Sigma}}}}}!}}$. Boltzmann’s equation of entropy would give $S_{{{\mathrm{d}}}} = k_{{{\mathrm{B}}}}ln\left( W \right) = k_{{{\mathrm{B}}}}ln\left( {\frac{{\left( {m_{{{{\mathrm{NO}}}}}^{{{\mathrm{{\Sigma}}}}} + m_{{{\mathrm{p}}}}^{{{\mathrm{{\Sigma}}}}}} \right)!}}{{m_{{{\mathrm{p}}}}^{{{\mathrm{{\Sigma}}}}}!m_{{{{\mathrm{NO}}}}}^{{{\mathrm{{\Sigma}}}}}!}}} \right)$. For a pure fluid, only one undistinguishable combination exists, i.e. *S*_f_ = *k*_B_*ln*(1) = 0. The corresponding surface fraction is given by $y_{{{\mathrm{p}}}} = \frac{{m_{{{\mathrm{p}}}}^{{{\mathrm{{\Sigma}}}}}}}{{m_{{{{\mathrm{NO}}}}}^{{{\mathrm{{\Sigma}}}}} + m_{{{\mathrm{p}}}}^{{{\mathrm{{\Sigma}}}}}}}$. The surface coverage is defined to be equal to the surface fraction, *θ*_i_ ≡ *y*_i_, so that *θ*_p_ + *θ*_NO_ = 1. Defining the configurational entropy of dispersing, due to nanoparticle coverage and non-occupied sites, as ∆*s*_d_ = *S*_d_*N*_A_ − *S*_f_*N*_A_, and using Stirling’s approximation for the logarithm of factorials, gives

9 $${\Delta}s_{{{\mathrm{d}}}}^{{{\mathrm{{\Sigma}}}}} = - k_{{{\mathrm{B}}}}N_{{{\mathrm{A}}}}\left( {\theta _{{{\mathrm{p}}}}ln\left( {\theta _{{{\mathrm{p}}}}} \right) + \theta _{{{{\mathrm{NO}}}}}ln\left( {\theta _{{{{\mathrm{NO}}}}}} \right)} \right)$$

where *k*_B_ and *N*_A_ are, respectively, Boltzmann’s constant and Avogadro’s number. In dilute conditions, the enthalpy of dispersing can be neglected. It should be noted that this enthalpy results from heat liberated or absorbed due to new interactions that stem from the mixing process, while it is not the same as the enthalpy of adsorption, which plays a role in the equilibrium adsorption constant. In such conditions, we deal with an ideal dispersion, being consistent with the Langmuir’s adsorption isotherm, of which a detailed deduction is presented in the next section. The Gibbs surface free energy of dispersing is then given by ${\Delta}g_{{{\mathrm{d}}}}^{{{\mathrm{{\Sigma}}}}} = - T{\Delta}s_{{{\mathrm{d}}}}^{{{\mathrm{{\Sigma}}}}}$ resulting into

10 $${\Delta}g_{{{\mathrm{d}}}}^{{{\mathrm{{\Sigma}}}}} = k_{{{\mathrm{B}}}}TN_{{{\mathrm{A}}}}\left( {\theta _{{{\mathrm{p}}}}ln\left( {\theta _{{{\mathrm{p}}}}} \right) + \theta _{{{{\mathrm{NO}}}}}ln\left( {\theta _{{{{\mathrm{NO}}}}}} \right)} \right)$$

We define $\omega _{{{\mathrm{p}}}} \equiv \frac{{\varsigma _{{{\mathrm{p}}}}}}{{\varsigma _{{{{\mathrm{NO}}}}}}}$ and $\omega _{{{\mathrm{f}}}} \equiv \frac{{\varsigma _{{{\mathrm{f}}}}}}{{\varsigma _{{{{\mathrm{NO}}}}}}}$. The chemical potentials of a component *i* are defined by

11 $$\eta _{{{\mathrm{i}}}}^{{{\mathrm{{\Sigma}}}}} = N_{{{\mathrm{A}}}}\frac{\partial }{{\partial m_{{{\mathrm{i}}}}^{{{\mathrm{{\Sigma}}}}}}}\left( {\frac{{m_{{{{\mathrm{NO}}}}}^{{{\mathrm{{\Sigma}}}}} + m_{{{\mathrm{p}}}}^{{{\mathrm{{\Sigma}}}}}}}{{N_{{{\mathrm{A}}}}}}{\Delta}g_{{{\mathrm{d}}}}^{{{\mathrm{{\Sigma}}}}}} \right)_{T,p,m_{\forall {{{\mathrm{j}}}} \ne {{{\mathrm{i}}}}}^{{{\mathrm{{\Sigma}}}}}}$$

with *i* = *p*, *f* and *j* = *p*, *f*. The number *ω*_p_ can also be understood as the number of adsorption sites per nanoparticle. We then use the aforementioned definition $\theta _{{{\mathrm{i}}}} = \frac{{m_{{{\mathrm{i}}}}^{{{\mathrm{{\Sigma}}}}}}}{{m_{{{{\mathrm{NO}}}}}^{{{\mathrm{{\Sigma}}}}} + m_{{{\mathrm{p}}}}^{{{\mathrm{{\Sigma}}}}}}}$, fill this in in Eq. (10), apply Eq. (11) and rewrite the result back in terms of *θ*_i_. This finally gives $\eta _{{{\mathrm{f}}}}^{{{\mathrm{{\Sigma}}}}} = k_{{{\mathrm{B}}}}TN_{{{\mathrm{A}}}}\omega _{{{\mathrm{f}}}}ln\left( {1 - \theta _{{{\mathrm{p}}}}} \right)$ and

12 $$\eta _{{{\mathrm{p}}}}^{{{\mathrm{{\Sigma}}}}} = k_{{{\mathrm{B}}}}TN_{{{\mathrm{A}}}}\left( {ln\left( {\theta _{{{\mathrm{p}}}}} \right) - \omega _{{{\mathrm{p}}}}ln\left( {1 - \theta _{{{\mathrm{p}}}}} \right)} \right)$$

where $\omega _{{{\mathrm{p}}}} \equiv \frac{{\varsigma _{{{\mathrm{p}}}}}}{{\varsigma _{{{{\mathrm{NO}}}}}}} \equiv \frac{{\varsigma _{{{\mathrm{p}}}}}}{{\varsigma _{{{\mathrm{f}}}}}}$ (as $\omega _{{{\mathrm{f}}}} \equiv \frac{{\varsigma _{{{\mathrm{f}}}}}}{{\varsigma _{{{{\mathrm{NO}}}}}}}$ and *ω*_f_ ≡ 1 because the adsorption sites are within the present framework geometrically equivalent to the projected liquid molecules, see Fig. 1b and corresponding discussion) is given by

13 $$\omega _{{{\mathrm{p}}}} = \frac{{M_{{{\mathrm{p}}}}\rho _{{{\mathrm{f}}}}{{{\mathcal{L}}}}_{{{\mathrm{f}}}}}}{{\rho _{{{\mathrm{p}}}}M_{{{\mathrm{f}}}}{{{\mathcal{L}}}}_{{{\mathrm{p}}}}}}$$

### Surface adsorption isotherm

Equilibrium of the adsorption process is described by a net zero change of the total Gibbs free energy of the system: ${{{\mathrm{d}}}}{\Delta}G_{{{\mathrm{a}}}} = d{\Delta}G_{{{{\mathrm{ad}}}}}^{{{\mathrm{{\Sigma}}}}} + d{\Delta}G_{{{{\mathrm{ad}}}}}^{{{\mathrm{b}}}} + d{\Delta}G_{{{\mathrm{d}}}}^{{{\mathrm{{\Sigma}}}}} + d{\Delta}G_{{{\mathrm{d}}}}^{{{\mathrm{b}}}} \equiv 0$, where the subscripts ‘*ad*’ and ‘*d*’ denote the adsorption (due to translational or confinement effects, or effects related to particle surface energies, dipole-dipole and coulomb interactions^24^, for instance) and the dispersion (mixing) free energies, respectively, and ‘Σ’ and ‘b’ the surface and bulk phases, respectively. We focus first on the dispersing. We can then define ${\Delta}G_{{{\mathrm{d}}}}^{{{\mathrm{{\Sigma}}}}} = A\frac{{m_{{{{\mathrm{NO}}}}}^{{{\mathrm{{\Sigma}}}}} + m_{{{\mathrm{p}}}}^{{{\mathrm{{\Sigma}}}}}}}{{N_{{{\mathrm{A}}}}}}{\Delta}g_{{{\mathrm{d}}}}^{{{\mathrm{{\Sigma}}}}}$ and ${\Delta}G_{{{\mathrm{d}}}}^{{{\mathrm{b}}}} = V\frac{{m_{{{\mathrm{l}}}}^{{{\mathrm{b}}}} + m_{{{\mathrm{p}}}}^{{{\mathrm{b}}}}}}{{N_{{{\mathrm{A}}}}}}{\Delta}g_{{{\mathrm{d}}}}^{{{\mathrm{b}}}}$, where $m_{{{\mathrm{l}}}}^{{{\mathrm{b}}}}$ and $m_{{{\mathrm{p}}}}^{{{\mathrm{b}}}}$ are the number of bulk fluid particles and nanoparticles per unit volume of the dispersion, whereas *A* and *V* are an arbitrary unit surface and volume, respectively. Note here that $\frac{{m_{{{{\mathrm{NO}}}}}^{{{\mathrm{{\Sigma}}}}} + m_{{{\mathrm{p}}}}^{{{\mathrm{{\Sigma}}}}}}}{{N_{{{\mathrm{A}}}}}}$ has unit moles per unit surface and $\frac{{m_{{{\mathrm{l}}}}^{{{\mathrm{b}}}} + m_{{{\mathrm{p}}}}^{{{\mathrm{b}}}}}}{{N_{{{\mathrm{A}}}}}}$ unit moles per unit volume. Note also that we can define a mole fraction of nanoparticles in the bulk as $x_{{{\mathrm{p}}}} = \frac{{m_{{{\mathrm{p}}}}^{{{\mathrm{b}}}}}}{{m_{{{\mathrm{l}}}}^{{{\mathrm{b}}}} + m_{{{\mathrm{p}}}}^{{{\mathrm{b}}}}}}$. Neglecting the enthalpy of dispersing as mentioned before, an addition of nanoparticles to the surface would result into a change ${{{\mathrm{d}}}}{\Delta}G_{{{\mathrm{d}}}}^{{{\mathrm{{\Sigma}}}}}\left( {m_{{{\mathrm{p}}}}^{{{\mathrm{{\Sigma}}}}},m_{{{\mathrm{f}}}}^{{{\mathrm{{\Sigma}}}}}} \right)$, which can mathematically be written as ${{{\mathrm{d}}}}{\Delta}G_{{{\mathrm{d}}}}^{{{\mathrm{{\Sigma}}}}} = N_{{{\mathrm{A}}}}\frac{\partial }{{\partial m_{{{\mathrm{p}}}}^{{{\mathrm{{\Sigma}}}}}}}\left( {A\frac{{m_{{{{\mathrm{NO}}}}}^{{{\mathrm{{\Sigma}}}}} + m_{{{\mathrm{p}}}}^{{{\mathrm{{\Sigma}}}}}}}{{N_{{{\mathrm{A}}}}}}{\Delta}g_{{{\mathrm{d}}}}^{{{\mathrm{{\Sigma}}}}}} \right)_{T,p,m_{{{\mathrm{f}}}}^{{{\mathrm{{\Sigma}}}}}}{{{\mathrm{d}}}}m_{{{\mathrm{p}}}}^{{{\mathrm{{\Sigma}}}}} + N_{{{\mathrm{A}}}}\frac{\partial }{{\partial m_{{{\mathrm{f}}}}^{{{\mathrm{{\Sigma}}}}}}}\left( {A\frac{{m_{{{{\mathrm{NO}}}}}^{{{\mathrm{{\Sigma}}}}} + m_{{{\mathrm{p}}}}^{{{\mathrm{{\Sigma}}}}}}}{{N_{{{\mathrm{A}}}}}}{\Delta}g_{{{\mathrm{d}}}}^{{{\mathrm{{\Sigma}}}}}} \right)_{T,p,m_{{{\mathrm{p}}}}^{{{\mathrm{{\Sigma}}}}}}{{{\mathrm{d}}}}m_{{{\mathrm{f}}}}^{{{\mathrm{{\Sigma}}}}} = A\left( {\eta _{{{\mathrm{p}}}}^{{{\mathrm{{\Sigma}}}}}{{{\mathrm{d}}}}m_{{{\mathrm{p}}}}^{{{\mathrm{{\Sigma}}}}} + \eta _{{{\mathrm{f}}}}^{{{\mathrm{{\Sigma}}}}}{{{\mathrm{d}}}}m_{{{\mathrm{f}}}}^{{{\mathrm{{\Sigma}}}}}} \right)$. From (3) and the total number density, it can be derived that ${{{\mathrm{d}}}}m_{{{\mathrm{f}}}}^{{{\mathrm{{\Sigma}}}}} = \left( {\omega _{{{\mathrm{p}}}} - 1} \right){{{\mathrm{d}}}}m_{{{\mathrm{p}}}}^{{{\mathrm{{\Sigma}}}}}$. With the definitions for $\eta _{{{\mathrm{p}}}}^{{{\mathrm{{\Sigma}}}}}$ and $\eta _{{{\mathrm{f}}}}^{{{\mathrm{{\Sigma}}}}}$ (see Eq. (12) and text above), this leads with *ω*_f_ = 1 to ${{{\mathrm{d}}}}{\Delta}G_{{{\mathrm{d}}}}^{{{\mathrm{{\Sigma}}}}} = k_{{{\mathrm{B}}}}TN_{{{\mathrm{A}}}}ln\left( {\frac{{\theta _{{{\mathrm{p}}}}}}{{1 - \theta _{{{\mathrm{p}}}}}}} \right)A\,{{{\mathrm{d}}}}m_{{{\mathrm{p}}}}^{{{\mathrm{{\Sigma}}}}}$. An equivalent procedure can be performed for the bulk phase leading, under the approximation of diluted dispersion, to the relation ${{{\mathrm{d}}}}{\Delta}G_{{{\mathrm{d}}}}^{{{\mathrm{b}}}} = k_{{{\mathrm{B}}}}TN_{{{\mathrm{A}}}}ln\left( {x_{{{\mathrm{p}}}}} \right)V\,{{{\mathrm{d}}}}m_{{{\mathrm{p}}}}^{{{\mathrm{b}}}}$. Mass conservation stipulates that the net mass change is zero, i.e. $A\,{{{\mathrm{d}}}}m_{{{\mathrm{p}}}}^{{{\mathrm{{\Sigma}}}}} + V\,{{{\mathrm{d}}}}m_{{{\mathrm{p}}}}^{{{\mathrm{b}}}} = 0$. This leads to ${{{\mathrm{d}}}}{\Delta}G_{{{\mathrm{d}}}}^{{{\mathrm{{\Sigma}}}}} + {{{\mathrm{d}}}}{\Delta}G_{{{\mathrm{d}}}}^{{{\mathrm{b}}}} = k_{{{\mathrm{B}}}}TN_{{{\mathrm{A}}}}( {ln( {\frac{{\theta _{{{\mathrm{p}}}}}}{{1 - \theta _{{{\mathrm{p}}}}}}} ) - ln( {x_{{{\mathrm{p}}}}})} )A\,{{{\mathrm{d}}}}m_{{{\mathrm{p}}}}^{{{\mathrm{{\Sigma}}}}}$.

A change in the nanoparticles number in both phases upon adsorption also induces a change in the free energy of the adsorption, which can be symbolically (as an already existing thermodynamic relation for the free energy of adsorption will be used, there is no need to enter into details as we did for the free energy of dispersion earlier) written as ${{{\mathrm{d}}}}{\Delta}G_{{{{\mathrm{ad}}}}}^{\Sigma}\left( {A\,m_{{{\mathrm{p}}}}^{\Sigma}} \right) + {{{\mathrm{d}}}}{\Delta}G_{{{{\mathrm{ad}}}}}^{{{\mathrm{b}}}}\left( {V\,m_{{{\mathrm{p}}}}^b} \right) = {\Delta}g_{{{{\mathrm{ad}}}}}^{\Sigma}A\,{{{\mathrm{d}}}}m_{{{\mathrm{p}}}}^{\Sigma} + {\Delta}g_{{{{\mathrm{ad}}}}}^{{{\mathrm{b}}}}V\,{{{\mathrm{d}}}}m_{{{\mathrm{p}}}}^b$. We can use the mass conservation principle $A\,{{{\mathrm{d}}}}m_{{{\mathrm{p}}}}^{\Sigma} + V\,{{{\mathrm{d}}}}m_{{{\mathrm{p}}}}^{{{\mathrm{b}}}} = 0$ and write ${{{\mathrm{d}}}}{\Delta}G_{{{{\mathrm{ad}}}}}^{\Sigma} + {{{\mathrm{d}}}}{\Delta}G_{{{{\mathrm{ad}}}}}^{{{\mathrm{b}}}} = \left( {{\Delta}g_{{{{\mathrm{ad}}}}}^{{{\mathrm{{\Sigma}}}}} - {\Delta}g_{{{{\mathrm{ad}}}}}^{{{\mathrm{b}}}}} \right)A\,{{{\mathrm{d}}}}m_{{{\mathrm{p}}}}^{\Sigma} \equiv {\Delta}g_{{{{\mathrm{ad}}}}}A\,{{{\mathrm{d}}}}m_{{{\mathrm{p}}}}^{\Sigma}$, where ∆*g*_ad_ stands for the net difference of the free energy of adsorption per mole of adsorbed nanoparticles. At equilibrium, ∆*g*_ad_ is related to the thermodynamic equilibrium constant *K*_e_ via Van‘t Hoff’s equation for adsorption ∆*g*_ad_ = −*RTln*(*K*_e_) with *K*_e_ the thermodynamic equilibrium constant of adsorption. The total Gibbs free energy of the system d∆*G*_a_ becomes finally ${{{\mathrm{d}}}}{\Delta}G_{{{\mathrm{a}}}} = k_{{{\mathrm{B}}}}TN_{{{\mathrm{A}}}}( {ln( {\frac{{\theta _{{{\mathrm{p}}}}}}{{1 - \theta _{{{\mathrm{p}}}}}}} ) - ln( {x_{{{\mathrm{p}}}}})} )A\,{{{\mathrm{d}}}}m_{{{\mathrm{p}}}}^{\Sigma} - RTln( {K_{{{\mathrm{e}}}}} )A\,{{{\mathrm{d}}}}m_{{{\mathrm{p}}}}^{\Sigma} \equiv 0$ at equilibrium. This leads finally to $- lnK_{{{\mathrm{e}}}} + ln\left( {\frac{{\theta _{{{\mathrm{p}}}}}}{{1 - \theta _{{{\mathrm{p}}}}}}} \right) - ln\left( {x_{{{\mathrm{p}}}}} \right) = 0$, which is known as an adsorption isotherm for ideal dispersions or solutions. The equilibrium constant *K*_e_ is for ideal cases related to the dimensional Langmuir equilibrium constant $K_{{{\mathrm{p}}}}^{{{\mathrm{d}}}}$, which can be traditionally described by the equilibrium adsorption reaction: *c*_p_ + [*] ⇆ [P − *], where *c*_p_ is the nanoparticle molar bulk concentration, [*] the surface molar concentration of empty adsorption sites and [*P* − *] the surface molar concentration of adsorbed nanoparticles, respectively. If we define ${\Gamma}_{{{{\mathrm{p}}}},{{{\mathrm{max}}}}}^{{{\mathrm{{\Sigma}}}}}$ as the maximum surface concentration, we can write $\left[ \ast \right] + \left[ {P - \ast } \right] = {\Gamma}_{{{{\mathrm{p}}}},{{{\mathrm{max}}}}}^{{{\mathrm{{\Sigma}}}}}$, which is equivalent to defining the surface coverage as $\theta _{{{\mathrm{p}}}} = \frac{{\left[ {P - \ast } \right]}}{{{\Gamma}_{{{{\mathrm{p}}}},{{{\mathrm{max}}}}}^{{{\mathrm{{\Sigma}}}}}}}$ and therefore $\frac{{\left[ \ast \right]}}{{{\Gamma}_{{{{\mathrm{p}}}},{{{\mathrm{max}}}}}^{{{\mathrm{{\Sigma}}}}}}} \equiv 1 - \theta _{{{\mathrm{p}}}}$. Note that later, we will use the notation *Γ*_p_^Σ^ for [*P* − *]. Thermodynamically, $K_{{{\mathrm{p}}}}^{{{\mathrm{d}}}} = \frac{{\left[ {P - \ast } \right]}}{{c_{{{\mathrm{p}}}}\left[ \ast \right]}} = \frac{{\theta _{{{\mathrm{p}}}}}}{{c_{{{\mathrm{p}}}}\left( {1 - \theta _{{{\mathrm{p}}}}} \right)}}$. As *c*_p_ has unity moles per unit volume, $K_{{{\mathrm{p}}}}^{{{\mathrm{d}}}}$ has unity volume per mole. Furthermore, as *K*_e_ is dimensionless, this means that we can define $K_{{{\mathrm{e}}}} = c_{{{\mathrm{t}}}}K_{{{\mathrm{p}}}}^{{{\mathrm{d}}}}$, where *c*_t_ must have unity moles per volume. Similar discussions on the various definitions of *K*_e_ and $K_{{{\mathrm{p}}}}^{{{\mathrm{d}}}}$ have been performed in the literature, indicating that *K*_e_ in Van’t Hoff’s equation is dimensionless and that $K_{{{\mathrm{p}}}}^{{{\mathrm{d}}}}$ in the Langmuir’s adsorption equation has a dimension depending on the concentrations, confirming this analysis^25,26^. We can also deduce (in dilute systems, *c*_f_ ≈ *c*_t_, with *c*_f_ and *c*_t_ the molar concentrations of the base fluid and the bulk phase, respectively) that *c*_t_ can be represented by the molar concentration of the bulk phase^25,26^. Filling this in the adsorption isotherm gives finally $- ln\left( {c_{{{\mathrm{t}}}}K_{{{\mathrm{p}}}}^{{{\mathrm{d}}}}} \right) + ln\left( {\frac{{\theta _{{{\mathrm{p}}}}}}{{1 - \theta _{{{\mathrm{p}}}}}}} \right) - ln\left( {x_{{{\mathrm{p}}}}} \right) = 0$, which (keeping in mind that *x*_p_*c*_t_ = *c*_p_) simplifies to $\frac{{\theta _{{{\mathrm{p}}}}}}{{1 - \theta _{{{\mathrm{p}}}}}} = K_{{{\mathrm{p}}}}^{{{\mathrm{d}}}}c_{{{\mathrm{p}}}}$, which is the well-known Langmuir’s adsorption isotherm, subject to further discussion in the next section. This can be rearranged into

14 $$\theta _{{{\mathrm{p}}}} = \frac{{K_{{{\mathrm{p}}}}^{{{\mathrm{d}}}}c_{{{\mathrm{p}}}}}}{{1 + K_{{{\mathrm{p}}}}^{{{\mathrm{d}}}}c_{{{\mathrm{p}}}}}}$$

The equilibrium adsorption constant *K*_e_ could be calculated thermodynamically via Van’t Hoff’s relation. However, experimental values for the molar adsorption enthalpies and entropies are not readily available for the studied nanoparticle dispersions and especially not for various concentrations. Other expressions and methods make use of more available surface energies and surface tensions. However, even if one may perform such measurements, such a procedure would not allow an analytical physics-based analysis of the behaviour of the surface tension and would not offer the understanding of the underlying mechanisms for the various surface tension behaviours. Therefore, it would not align with the purposes of this work. In order to obtain theoretical parameters, independent of the experimental surface tension data, experimental regression procedures or any fitting methods, one of the often-used ways is to use a kinetic model. Adsorption and desorption are often described kinetically. Material properties for kinetic models are readily available for solid–liquid interfaces and the methods are widely used and understood. As less data are available for liquid–fluid interfaces, it is the question whether similar kinetic models would be applicable. One can argue that the adsorption of surface-charged nanoparticles (an important method to obtain stable dispersions) on liquid-fluid interfaces (often charged with the same sign) can be approximated by adsorption on solid–liquid interfaces. Although subject to more investigation, it has already been applied successfully for liquid–fluid interfaces^24^. This motivates that within such a reasonable assumption the equilibrium adsorption constant for the nanoparticle dispersions can be obtained without fitting. The interpretation of underlying mechanisms would benefit from such a physics-based approach.

### Surface kinetics

It remains to find the equilibrium adsorption constant $K_{{{\mathrm{p}}}}^{{{\mathrm{d}}}}$ or for later convenience, a dimensionless version *K*_p_ thereof. ‘*Surface adsorption isotherm*’ presented the thermodynamic theory behind the Langmuir adsorption isotherm. It was mentioned that unavailable experimental data for the nanoparticle dispersions studied here and the aim to provide for a physics-based model encourage the use of another way. Commonly, the equilibrium adsorption constant is determined kinetically, where material properties necessary for the model are readily available. The kinetic model is based on an equilibrium between standard adsorption and desorption kinetics and is treated in details in the literature^27–30^. We mention the main points here. Note that desorption becomes relevant when the energy of particle trapping is of the order of the thermal energy. Adsorption (with standard rate *k*_a_) depends on the bulk concentration *c*_p_ and the available adsorption sites (1 − *θ*_p_). Desorption (with standard rate *k*_d_) depends on the adsorbed nanoparticles *θ*_p_ per specific surface area of adsorbed nanoparticles *ς*_p_. This writes as

15 $$j_{{{\mathrm{a}}}} = k_{{{\mathrm{a}}}}c_{{{\mathrm{p}}}}\left( {1 - \theta _{{{\mathrm{p}}}}} \right)$$

16 $$j_{{{\mathrm{d}}}} = k_{{{\mathrm{d}}}}\theta _{{{\mathrm{p}}}}\frac{1}{{\varsigma _{{{\mathrm{p}}}}}}$$

From kinetic considerations, it can be stated that nanoparticle accumulation, through a flux balance equation, at the interface is given by $\frac{1}{{\varsigma _{{{\mathrm{p}}}}}}\frac{{\partial \theta _{{{\mathrm{p}}}}}}{{\partial t}} = j_{{{\mathrm{a}}}} - j_{{{\mathrm{d}}}}$, where we remind that here *ς*_p_ is the specific surface area per *mole* of nanoparticles. At quasi-stationarity, i.e. $\frac{{\partial \theta _{{{\mathrm{p}}}}}}{{\partial t}} = 0$, we have from Eqs. (15) and (16) that

17 $$\theta _{{{\mathrm{p}}}} = \frac{{k_{{{\mathrm{a}}}}c_{{{\mathrm{p}}}}}}{{k_{{{\mathrm{d}}}}\frac{1}{{\varsigma _{{{\mathrm{p}}}}}} + k_{{{\mathrm{a}}}}c_{{{\mathrm{p}}}}}} = \frac{{\frac{{k_{{{\mathrm{a}}}}}}{{k_{{{\mathrm{d}}}}}}\varsigma _{{{\mathrm{p}}}}c_{{{\mathrm{p}}}}}}{{1 + \frac{{k_{{{\mathrm{a}}}}}}{{k_{{{\mathrm{d}}}}}}\varsigma _{{{\mathrm{p}}}}c_{{{\mathrm{p}}}}}}$$

Comparison with (14) learns that $K_{{{\mathrm{p}}}}^{{{\mathrm{d}}}} = \frac{{k_{{{\mathrm{a}}}}}}{{k_{{{\mathrm{d}}}}}}\varsigma _{{{\mathrm{p}}}}$. As the molar concentration can also be expressed into the volume fraction *φ* by $c_{{{\mathrm{p}}}} \equiv \frac{{\rho _{{{\mathrm{p}}}}}}{{M_{{{\mathrm{p}}}}}}\varphi$, we can rewrite (17) as

18 $$\theta _{{{\mathrm{p}}}} = \frac{{\frac{{k_{{{\mathrm{a}}}}}}{{k_{{{\mathrm{d}}}}}}\frac{{\rho _{{{\mathrm{p}}}}}}{{M_{{{\mathrm{p}}}}}}\varsigma _{{{\mathrm{p}}}}\varphi }}{{1 + \frac{{k_{{{\mathrm{a}}}}}}{{k_{{{\mathrm{d}}}}}}\frac{{\rho _{{{\mathrm{p}}}}}}{{M_{{{\mathrm{p}}}}}}\varsigma _{{{\mathrm{p}}}}\varphi }}$$

This allows defining a dimensionless Langmuir’s equilibrium constant $K_{{{\mathrm{p}}}} \equiv \frac{{k_{{{\mathrm{a}}}}}}{{k_{{{\mathrm{d}}}}}}\frac{{\rho _{{{\mathrm{p}}}}}}{{M_{{{\mathrm{p}}}}}}\varsigma _{{{\mathrm{p}}}} = \frac{{k_{{{\mathrm{a}}}}}}{{k_{{{\mathrm{d}}}}}}\frac{1}{{{{{\mathcal{L}}}}_{{{\mathrm{p}}}}}}$, and relating the surface coverage with the bulk volume fraction as

19 $$\theta _{{{\mathrm{p}}}} = \frac{{K_{{{\mathrm{p}}}}\varphi }}{{1 + K_{{{\mathrm{p}}}}\varphi }}$$

If we assume that particle transport occurs under a quasi-linear and stationary diffusional regime (this is a valid approximation because of the very small relaxation time), it has been shown that general analytical solutions for the adsorption and desorption constants can be obtained^27–30^, i.e. $\frac{{k_a}}{{k_d}} = \delta _m\sqrt {\frac{{\pi k_BT}}{{\left| {{\Phi}_m} \right|}}} e^{ - \frac{{{\Phi}_m}}{{k_BT}}}$, which finally leads to

20 $$K_p \equiv \frac{{\delta _m}}{{{{{\mathcal{L}}}}_p}}\sqrt {\frac{{\pi k_BT}}{{\left| {{\Phi}_m} \right|}}} e^{ - \frac{{{\Phi}_m}}{{k_BT}}}$$

where *Φ*_m_ is the total potential energy, *Φ*_t_, at a distance *z* = *δ*_m_, i.e. ${{{\mathrm{{\Phi}}}}}_{{{\mathrm{m}}}} = \left. {{{{\mathrm{{\Phi}}}}}_t} \right|_{z = {\updelta}_{{{\mathrm{m}}}}}$. The total potential energy stands here for the potential energy between a particle and the liquid-air interface, being composed of many mechanisms. The DLVO theory mentions that the most important interactions are the electrostatic $\left( {{{{\mathrm{{\Phi}}}}}_{{{{\mathrm{p}}}} - {{{\mathrm{{\Sigma}}}}}}^{{{{\mathrm{EDL}}}}}} \right)$ and van der Waals $\left( {{{{\mathrm{{\Phi}}}}}_{{{{\mathrm{p}}}} - {{{\mathrm{{\Sigma}}}}}}^{{{{\mathrm{vdW}}}}}} \right)$ interaction energies^24,31–33^. Image charge effects in the form of a repulsive particle-image $\left( {{{{\mathrm{{\Phi}}}}}_{{{{\mathrm{p}}}} - {{{\mathrm{p}}}}\prime }^{{{{\mathrm{EDL}}}}}} \right)$ potential energy are esteemed to be of importance, the reason being that in cases of particles being oppositely charged to the interface, repulsion was still observed^24,34^. Non-DLVO interaction energies $\left( {{{{\mathrm{{\Phi}}}}}_{{{{\mathrm{p}}}} - {{{\mathrm{{\Sigma}}}}}}^{{{{\mathrm{Hy}}}}}} \right)$, suggested to be of the Lewis acid-base type, also appear to be of great importance, such as hydrophilic repulsive interactions and hydrophobic attraction energies^35–38^. The electrostatic double layer interaction potential between a nanoparticle and a flat fluid–air interface is given by^32^

21 $${{{\mathrm{{\Phi}}}}}_{{{{\mathrm{p}}}} - {{{\mathrm{{\Sigma}}}}}}^{{{{\mathrm{EDL}}}}} = 64{\uppi}\varepsilon _{{{\mathrm{r}}}}\varepsilon _0\left( {\frac{{k_{{{\mathrm{B}}}}T}}{{e_{{{\mathrm{e}}}}}}} \right)^2{{{\mathrm{Tanh}}}}\left( {\frac{{\zeta _{{{\mathrm{p}}}}e_{{{\mathrm{e}}}}}}{{4k_{{{\mathrm{B}}}}T}}} \right){{{\mathrm{Tanh}}}}\left( {\frac{{\zeta _{{{\mathrm{{\Sigma}}}}}e_{{{\mathrm{e}}}}}}{{4k_{{{\mathrm{B}}}}T}}} \right)\left( {a_{{{\mathrm{p}}}}\left( {{{{\mathrm{e}}}}^{ - \frac{z}{{\lambda _{{{\mathrm{D}}}}}}} + {{{\mathrm{e}}}}^{ - \frac{{z + 2a_{{{\mathrm{p}}}}}}{{\lambda _{{{\mathrm{D}}}}}}}} \right) + \lambda _{{{\mathrm{D}}}}\left( { - {{{\mathrm{e}}}}^{ - \frac{z}{{\lambda _{{{\mathrm{D}}}}}}} + {{{\mathrm{e}}}}^{ - \frac{{z + 2a_{{{\mathrm{p}}}}}}{{\lambda _{{{\mathrm{D}}}}}}}} \right)} \right)$$

where *ε*_r_, *ε*_0_, *e*_e_, *ζ*_p_, *ζ*_Σ_, and *λ*_D_, are, respectively, the relative permittivity, the absolute permittivity, the elementary charge, the zeta potential of the nanoparticles, the zeta potential of the liquid–air interface and Debye length. Debye’s length is given by $\lambda _{{{\mathrm{D}}}} = \sqrt {\frac{{\varepsilon _{{{\mathrm{r}}}}\varepsilon _0k_{{{\mathrm{B}}}}T}}{{2N_{{{\mathrm{A}}}}e_{{{\mathrm{e}}}}^2I}}}$, where *I* stands for the ionic strength of the base fluid. The potential energy between a particle, *p*, and its image, *p*′, in the phase at the other side of the fluid-air interface is given by refs. ^24,34^

22 $${{{\mathrm{{\Phi}}}}}_{{{{\mathrm{p}}}} - {{{\mathrm{p}}}}\prime }^{{{{\mathrm{EDL}}}}} = 32{\uppi}\varepsilon _{{{\mathrm{r}}}}\varepsilon _0\left( {\frac{{k_{{{\mathrm{B}}}}T}}{{e_{{{\mathrm{e}}}}}}} \right)^2{{{\mathrm{Tanh}}}}\left( {\frac{{\zeta _{{{\mathrm{p}}}}e_{{{\mathrm{e}}}}}}{{4k_{{{\mathrm{B}}}}T}}} \right){{{\mathrm{Tanh}}}}\left( {\frac{{\zeta _{{{{\mathrm{p}}}}\prime }e_{{{\mathrm{e}}}}}}{{4k_{{{\mathrm{B}}}}T}}} \right)a_{{{\mathrm{p}}}}{{{\mathrm{e}}}}^{ - 2\frac{z}{{\lambda _{{{\mathrm{D}}}}}}}$$

where *ζ*_p′_ stands for the zeta potential of the image particle, given by $\zeta _{{{{\mathrm{p}}}}^\prime } = \frac{{2k_{{{\mathrm{B}}}}T}}{{e_{{{\mathrm{e}}}}}}{{{\mathrm{ArcSinh}}}}( {\frac{{\varepsilon _{{{\mathrm{r}}}} - \varepsilon _{{{{\mathrm{r}}}}^\prime }}}{{\varepsilon _{{{\mathrm{r}}}} + \varepsilon _{{{{\mathrm{r}}}}^\prime }}}{{{\mathrm{Sinh}}}}( {\frac{{\zeta _{{{\mathrm{p}}}}e_{{{\mathrm{e}}}}}}{{2k_{{{\mathrm{B}}}}T}}} )} )$^33^. Here, *ε*_r′_ is the relative permittivity of the phase at the opposite side of the interface opposed to the relative permittivity of the phase where the nanoparticles are dispersed, *ε*_r_. The van der Waals potential energy between the nanoparticle and the interface is given by

23 $${{{\mathrm{{\Phi}}}}}_{{{{\mathrm{p}}}} - {{{\mathrm{{\Sigma}}}}}}^{{{{\mathrm{vdW}}}}} = - \frac{{A_{{{{\mathrm{p}}}} - {{{\mathrm{{\Sigma}}}}}}}}{6}\left( {\frac{{a_{{{\mathrm{p}}}}}}{z} + \frac{{a_{{{\mathrm{p}}}}}}{{z + 2a_{{{\mathrm{p}}}}}} + ln\left( {\frac{z}{{z + 2a_{{{\mathrm{p}}}}}}} \right)} \right)$$

where *A*_p−Σ_ is the non-retarded Hamaker constant for the particle–interface interaction, where the particle (*p*) interaction with air (*a*) through the base fluid (*f*) is assessed. This constant is derived by the theory of London-dispersion forces and is often approximated by the combining relation $A_{{{{\mathrm{p}}}} - {{{\mathrm{{\Sigma}}}}}} = A_{{{{\mathrm{pfa}}}}} = \left( {\sqrt {A_{{{{\mathrm{pp}}}}}} - \sqrt {A_{{{{\mathrm{ff}}}}}} } \right)\left( {\sqrt {A_{{{{\mathrm{aa}}}}}} - \sqrt {A_{{{{\mathrm{ff}}}}}} } \right)$^39,40^. Non-DLVO interaction energies may be considered as one potential energy, being either repulsive or attractive depending on the solid–water contact angle^36^. In another work, a Hydra parameter, depending on the hydrophobicity of the surface, was introduced in one expression, being either negative or positive, defining, respectively, a hydrophilic repulsive or hydrophobic attractive potential energy. The Hydra potential energy is given by refs. ^36,37,41,42^

24 $${\Phi}_{p - {\Sigma}}^{Hy} = - 2\pi a_p\lambda _0\gamma _0\left( {1 - Cos\left( \vartheta \right)} \right)e^{\frac{{z_0 - z}}{{\lambda _0}}}$$

where *λ*_0_ is a decay length, *ϑ* the radial liquid–solid static contact angle and *z*_0_ a constant of the value of 0.16 nm^41,42^. The total potential energy is given by ${\Phi}_t = {\Phi}_{p - {\Sigma}}^{EDL} + {\Phi}_{p - p\prime }^{EDL} + {\Phi}_{p - {\Sigma}}^{vdW} + {\Phi}_{p - {\Sigma}}^{Hy}$.

### Material properties

Table 1 shows the material properties of the used nanodispersions at ambient temperature. Effect of temperature on solid properties is neglected. For the base fluids, only the densities are adapted for temperatures different than ambient. Since these values are well tabulated, not more attention is given. Some data are reported in the literature as a function of the mass fraction. If *ξ* is the nanoparticle mass fraction, *ρ*_p_ the nanoparticle density and *ρ*_*f*_ the fluid density, then the nanoparticle volume fraction *φ* can be calculated as $\varphi = \frac{\xi }{{\rho _{{{\mathrm{p}}}}}}\left( {\frac{\xi }{{\rho _{{{\mathrm{p}}}}}} + \frac{{1 - \xi }}{{\rho _{{{\mathrm{f}}}}}}} \right)^{ - 1}$. Table 1 also shows general physical constants used in the model.

**Table 1:** Material properties and physical constants.

Component^a^	Density [$kg\,m^{ - 3}$]	Molar mass [$kg\,mol^{ - 1}$]
Al_2_O_3_	$3950$	$0.102$
Al	$2700$	$0.027$
B	$2370$	$0.011$
MgO	$3580$	$0.040$
SiO_2_	$2650$	$0.060$
Ag	$10490$	$0.108$
Laponite	$2530$	$2.287$
ZnO	$5610$	$0.0814$
Dodecanethiol-ligated Au^b^	$4720$	$0.198$
Water (W)	$997$	$0.018$
n-decane (D)	$730$	$0.142$
Ethanol	$789$	$0.046$
Ethylene glycol (EG)	$1110$	$0.062$
Tri-ethylene glycol (TEG)	$1120$	$0.150$
n-dodecane (DD)	$750$	$0.170$
n-hexadecane (HD)	$773$	$0.226$

^a^First nine rows concern the nanoparticle densities *ρ*_p_ and molar masses *M*_p'_. The tenth to sixteenth-row concern the base fluid densities *ρ*_f_ and molar mases *M*_f_.

^b^Volume-averaged and mole-averaged values are given for the density and molecular weight, respectively, based on the dimensions of the core gold nanoparticle and the dodecanethiol ligand shell. Note that the molar mass *M*_*p*_*'* given here is the one of an atom or molecule. To obtain the molar mass of a nanoparticle, one must make the conversion $M_{{{\mathrm{p}}}} = M_{{{{\mathrm{p}}}}^\prime }f_{{{\mathrm{p}}}}\frac{{V_{{{\mathrm{p}}}}}}{{V_{{{{\mathrm{p}}}}^\prime }}} = \frac{{4{\uppi}^2}}{{9\sqrt 2 }}a_{{{\mathrm{p}}}}^3\rho _{{{\mathrm{p}}}}N_{{{\mathrm{A}}}}$.

The values of the used physical constants are *N*_*A*_ = 6.02 * 10^23^ [mol^−1^], *R*_*g*_ = 8.3145 [J mol^−1^ K^−1^], *ε*_0_ = 8.854*10^−12^ [C V^−1^ m^−1^], *e*_*e*_ = 1.602 * 10^−19^ [C] and *k*_*B*_ = 1.38 * 10^−23^ [J K^−1^].

Equations (20)–(24) allow calculating the equilibrium constant *K*_p_. Several data are needed for this calculation. These data are collected from the literature and tabulated in Table 2. A summary of the variables and their meaning is given in Table 3.

**Table 2:** Data needed for calculation of equilibrium constant *K*_p_.

NP-L	*A*_pp_ [10^−20^ J]	*A*_ff_ [10^−20^ J]	*ε*_*r*_ [−]	*ϑ* [°]	*λ*_0_ [nm]	−*ζ*_p_ [mV]	*T* [K]	2*a*_p_ [nm]	Ref.
Al_2_O_3_-W	15^a^	3.7^b^	80^c^	35^d^	0.72	64	300	50^w^	^6^
Al_2_O_3_-D	15^a^	5.45^b^	2^e^	26^f^	1.16	55	300	50^w^	^6^
Al-D	15^g^	5.45^b^	2^e^	33^h^	0.87	57	300	18	^6^
B-D	6.23^i^	5.45^b^	2^e^	33^h^	0.35	55	300	46^x^	^6^
Al_2_O_3_-E	15^a^	4.2^b^	25.3^i^	23^j^	1.83	38	300	50^w^	^6^
Al-E	15^g^	4.2^b^	25.3^i^	36^k^	0.98	61	300	18	^6^
B-E	6.23^l^	4.2^b^	25.3^i^	36^k^	0.71	63	300	46^x^	^6^
Al_2_O_3_-TEG	15^a^	5.8^b^	23.3^m^	30^n^	0.8	59	298	20	^10^
MgO-TEG	12.1^o^	5.8^b^	23.3^m^	30^n^	0.67	46	298	20	^10^
SiO_2_-W	6.5^a^	3.7^b^	80^c^	20.7^p^	0.8	50	298	30	^9^
Ag-W	50^g^	3.7^b^	80^c^	40^q^	0.78	45	298	100	^9^
Lap-W	1.06^r^	3.7^b^	80^c^	24^s^	0.57	49	300	25 (1.5)^y^	^19^
ZnO-EG	9.2^a^	5.6^b^	40^t^	36.4^u^	0.57	60	300	67	^11^
(dl)Au-D	28^v^	5.45^b^	2^e^	30.5^v^	1.95	30	303	5 (1.7)^z^	^38,47^
(dl)Au-DD	28^v^	5.8^b^	2^e^	33^v^	1.71	35	303	5 (1.7)^z^	^38,47^
(dl)Au-HD	28^v^	5.2^b^	2^e^	36^v^	1.5	70	303	5 (1.7)^z^	^38,47^
Al_2_O_3_-Ws	15^a^	3.7^b^	80^c^	35^d^	0.72	75	300	40	^48^

The base fluids W, D, DD, HD E, TEG, and EG stand for water, n-decane, n-dodecane, n-hexadecane, ethanol, tri-ethylene glycol, and ethylene glycol, respectively. Ws stands for fully stabilised water dispersion^48^. The temperatures for which the experimental data are obtained from the literature are indicated in the table. If in the literature it is mentioned that the experimental data are obtained at ambient temperature, the value of 300 K is used.

^a^ref. ^52^.

^b^ref. ^53^, the value of TEG is approximated as that of di-ethylene glycol.

^c^ref. ^54^.

^d^ref. ^55^.

^e^ref. ^56^, same value assumed for n-dodecane and n-hexadecane.

^f^ref. ^57^.

^g^ref. ^58^.

^h^ref. ^59^, the value of B is approximated as that of Al.

^i^ref. ^60^.

^j^refs. ^60–63^, interpolative estimation.

^k^ref. ^64^, the value of B is approximated as that of Al.

^l^ref. ^65^.

^m^ref. ^66^.

^n^ref. ^67^, assumed from values of EG on mixed ceramic substrates.

^o^ref. ^68^.

^p^ref. ^69^.

^q^refs. ^70,71^, averaged value.

^r^ref. ^72^.

^s^refs. ^73,74^, averaged values.

^t^refs. ^75,76^, averaged values.

^u^ref. ^77^, approximated.

^v^ref. ^38^.

^w^TEM images in ref. ^6^ show agglomeration so that the size of the nanoparticles is ~2 times that of the initial one (25 nm).

^x^SEM images in ref. ^6^ show cubic-like particles with an averaged size of 80 nm so that, taking this size between opposite corners of a cube, one cube side would be 80/√3≈46 nm.

^y^The value between the brackets is the thickness of the nanodisks.

^z^The core diameter of Au is 5 nm andthe ligand shell thickness is 1.7 nm withan overall reported diameter of 2*a*_p_ = 8.4 nm^38,47^. The B nanoparticles were approximated as cubic particles, evidenced from SEM images in ref. ^6^ and the Laponite nanoparticles are nanodisks of a flat (the thickness is much smaller than the radius) cylindrical shape^19^, while the rest are spherical nanoparticles^6,7,9–11,38,47,48^.

**Table 3:** Variables used in the model and their meaning.

Symbol	Description	Unit
*a*_p_	Nanoparticle radius	[m]
A_p−Σ_	Non-retarded Hamaker constant	[J]
*c*_p_	Bulk concentration	[mol m^−3^]
${{{\mathcal{D}}}}_{{{\mathrm{p}}}}$	Ratio excess surface to surface-equivalent of bulk concentrations	[−]
*e*_e_	Elementary charge	[C]
*f*_∞_	Maximum geometric coverage fraction	[−]
*h*_p_	Disk nanoparticle thickness	[m]
${{{\mathcal{H}}}}_{{{{\mathrm{NO}}}}}$	Non-occupancy effect	[J m^−2^]
*I*	Ionic strength	[mol m^−3^]
*k*_a_	Adsorption rate	[m s^−1^]
*k*_B_	Boltzmann constant	[J K^−1^]
*k*_d_	Desorption rate	[s^−1^]
*K*_p_	Equilibrium adsorption constant	[−]
*K*_Σ_	Ratio surface to bulk preference	[−]
$\ell _{{{\mathrm{f}}}}$	Equivalent size of fluid molecule	[m]
$\ell _{{{\mathrm{p}}}}$	Equivalent size of nanoparticle	[m]
${{{\mathcal{L}}}}_{{{\mathrm{f}}}}$	Characteristic length of fluid molecule	[m]
${{{\mathcal{L}}}}_{{{\mathrm{p}}}}$	Characteristic length of nanoparticle	[m]
$m_{{{\mathrm{f}}}}^{{{\mathrm{{\Sigma}}}}}$	Number of fluid molecules per unit surface	[particles m^−2^]
$m_{{{{\mathrm{NO}}}}}^{{{\mathrm{{\Sigma}}}}}$	Number of non-occupied sites per unit surface	[particles m^−2^]
$m_{{{\mathrm{p}}}}^{{{\mathrm{{\Sigma}}}}}$	Number of nanoparticles per unit surface	[particles m^−2^]
*M*_f_	Molar mass fluid molecule	[kg mol^−1^]
*M*_p_	Molar mass nanoparticle molecule unit	[kg mol^−1^]
*M*_p'_	Molar mass nanoparticle	[kg mol^−1^]
*n*	Normal vector	[−]
*N*_A_	Avogadro’s number	[particles mol^−1^]
*R*_g_	Universal gas constant	[J mol^−1^ K^−1^]
*T*	Temperature	[K]
Greek symbol		
*γ*	Surface tension	[N m^−1^]
*γ*_f_	Surface tension of fluid	[N m^−1^]
*γ*_T_	Temperature derivative of the surface tension	[N m^−1^ K^−1^]
*γ*_φ_	Volume-fraction derivative of the surface tension	[N m^−1^]
*Γ*_f_	Excess surface concentration of fluid	[mol m^−2^]
*Γ*_p_	Excess surface concentration of nanoparticles	[mol m^−2^]
*Γ*_p_^Σ^	Surface concentration of nanoparticles	[mol m^−2^]
*Γ*_b_^Σ^	Surface-equivalent of bulk concentration	[mol m^−2^]
${\Gamma}_{{{{\mathrm{p}}}},{{{\mathrm{max}}}}}^{{{\mathrm{{\Sigma}}}}}$	Maximum surface concentration	[mol m^−2^]
*δ*_m_	First minimum of potential well	[m]
*ε*_0_	Absolute electric permittivity	[C V^−1^ m^−1^]
*ε*_r_	Relative electric permittivity	[−]
*ζ*_int_	Zeta-potential interface	[V]
*ζ*_p_	Zeta-potential nanoparticles	[V]
$\eta _{{{\mathrm{f}}}}^{{{\mathrm{{\Sigma}}}}}$	Surface chemical potential fluid	[J mol^−1^]
$\eta _{{{\mathrm{p}}}}^{{{\mathrm{{\Sigma}}}}}$	Surface chemical potential nanoparticles	[J mol^−1^]
*θ*_NO_	Non-occupied site coverage	[−]
*θ*_p_	Nanoparticle coverage	[−]
*ϑ*	Contact angle	[°]
*λ*_0_	Decay length in hydra potential	[m]
*λ*_D_	Debye’s length	[m]
*ρ*_f_	Density of fluid	[kg m^−3^]
*ρ*_p_	Density of nanoparticles	[kg m^−3^]
*σ*_g_	Stress tensor gas-side of interface	[N m^−2^]
*σ*_l_	Stress tensor liquid-side of interface	[N m^−2^]
*ς*_f_	Specific surface area of fluid molecule	[m^2^ per particle]
*ς*_NO_	Specific surface area of non-occupied site	[m^2^ per particle]
*ς*_p_	Specific surface area of nanoparticle	[m^2^ per particle]
*φ*	Volume fraction	[−]
*Φ*_m_	Total potential energy at first minimum	[J]
${{{\mathrm{{\Phi}}}}}_{{{{\mathrm{p}}}} - {{{\mathrm{p}}}}\prime }^{{{{\mathrm{EDL}}}}}$	Repulsive particle-image potential energy	[J]
${{{\mathrm{{\Phi}}}}}_{{{{\mathrm{p}}}} - {{{\mathrm{{\Sigma}}}}}}^{{{{\mathrm{EDL}}}}}$	Electrostatic particle-interface potential energy	[J]
${{{\mathrm{{\Phi}}}}}_{{{{\mathrm{p}}}} - {{{\mathrm{{\Sigma}}}}}}^{{{{\mathrm{Hy}}}}}$	Hydra potential energy	[J]
${{{\mathrm{{\Phi}}}}}_{{{{\mathrm{p}}}} - {{{\mathrm{{\Sigma}}}}}}^{{{{\mathrm{vdW}}}}}$	Van der Waals potential energy	[J]
*Φ*_t_	Total potential energy	[J]
*ω*_p_	Number of adsorption sites for one nanoparticle	[−]
Subscript		
*p*	Nanoparticle	
NO	Non-occupied site	
*f*	Fluid	

It should be noted that it is difficult to obtain precise values for the parameters *ϑ*, *δ*_m_, *ζ*_int_, *I*, *λ*_0_, and *ζ*_p_, which need some discussion. Reasonable values can be obtained from experimental data for *ϑ*, *δ*_m_, *ζ*_int_, *I*. The minimum thickness between the nanoparticle and the interface at adsorption, *δ*_m_, is often assumed to be of the order of *δ*_m_ = 0.5 nm^27,43^. For the interface zeta potential, *ζ*_int_, the approximated mean value of *ζ*_int_ = −40 mV is taken for water^44,45^. For ethanol, tri-ethylene glycol, ethylene glycol and glycerol the same value is assumed, while n-decane, n-dodecane and n-hexadecane are considered to be an oily liquid as hexane and a value of *ζ*_int_ = −10 mV is taken^44^. The ionic strength of a fluid is somewhat an unknown. However, works have indicated that for deionized water, typical ionic strength values are measured of the order of *I* = 1 mol/m^3^^46^. This value is assumed for all the fluids used. The values for *λ*_0_ and *ζ*_p_ depend strongly on the experimental conditions and only ranges can be indicated. Decay lengths, *λ*_0_, of values up to 2.2 nm are reported for several systems^20,37,41^. The zeta-potentials *ζ*_p_ of nanodispersions were typically found to be approximately between −75 and −25 mV^20,34,43^. Educated guesses, not affecting the analysis in this work, for these two parameters within these indicated ranges are implemented in Table 4 for the calculation of the equilibrium constant. The obtained equilibrium constants for the nanoparticle dispersions are shown later in Table 5.

**Table 4:** Definitions, necessary for the calculation of the surface tension.

Symbol	Definition^a,b^			
${{{\mathcal{L}}}}_{{{\mathrm{p}}}}$	$\frac{{2a_{{{\mathrm{p}}}}}}{3}$	$\frac{{2a_{{{\mathrm{p}}}}}}{3}$	$\frac{{a_{{{\mathrm{p}}}}h_{{{\mathrm{p}}}}}}{{a_{{{\mathrm{p}}}} + h_{{{\mathrm{p}}}}}}$	−
*f*_∞_	0.547	1	0.907	−
${{{\mathcal{L}}}}_{{{\mathrm{f}}}}$	−	−	−	$\frac{{\ell _{{{\mathrm{f}}}}}}{3}$
*M*_p_	$\frac{{4{\uppi}^2}}{{9\sqrt 2 }}a_{{{\mathrm{p}}}}^3\rho _{{{\mathrm{p}}}}N_{{{\mathrm{A}}}}$			
${\Gamma}_{{{{\mathrm{p}}}},{{{\mathrm{max}}}}}^{{{\mathrm{{\Sigma}}}}}$	$f_\infty {{{\mathcal{L}}}}_{{{\mathrm{p}}}}\frac{{\rho _{{{\mathrm{p}}}}}}{{M_{{{\mathrm{p}}}}}}$			
*Γ*_b_^Σ^	$\frac{{\rho _{{{\mathrm{p}}}}}}{{M_{{{\mathrm{p}}}}}}\frac{{{{{\mathcal{L}}}}_{{{\mathrm{p}}}}^2}}{{{{{\mathcal{L}}}}_{{{\mathrm{f}}}}}}$			
*ω*_p_	$\frac{{M_{{{\mathrm{p}}}}\rho _{{{\mathrm{f}}}}{{{\mathcal{L}}}}_{{{\mathrm{f}}}}}}{{\rho _{{{\mathrm{p}}}}M_{{{\mathrm{f}}}}{{{\mathcal{L}}}}_{{{\mathrm{p}}}}}}$			
*K*_Σ_	$\frac{{{\Gamma}_{{{{\mathrm{p}}}},{{{\mathrm{max}}}}}^{{{\mathrm{{\Sigma}}}}}}}{{{\Gamma}_{{{\mathrm{b}}}}^{{{\mathrm{{\Sigma}}}}}}}$			
*K*_p_	$\frac{{{\updelta }}_{{{\mathrm{m}}}}}{{{{{\mathcal{L}}}}_{{{\mathrm{p}}}}}}\sqrt {\frac{{\pi k_{{{\mathrm{B}}}}T}}{{\left| {{\Phi}_{{{\mathrm{m}}}}} \right|}}} e^{ - \frac{{{\Phi}_{{{\mathrm{m}}}}}}{{k_{{{\mathrm{B}}}}T}}}$			

^a^For the first two rows, the first column stands for spherical nanoparticles, the second column for cubical nanoparticles and the third column for disk-like nanoparticles, while for the third row only the fourth column is used, standing for the fluid molecules.

^b^For the fourth to ninth rows, the definitions are general for all nanoparticle shapes and fluid molecules. The symbol *a*_p_ stands either for the radius of a spherical nanoparticle, half of the side of a cubical nanoparticle or the radius of a disk nanoparticle, the latter of which has thickness *h*_p_. Further, $\ell _{{{\mathrm{f}}}}$ is the equivalent radius of a sphere corresponding to the volume of a fluid molecule, while *ρ*_p_, *ρ*_f_, *M*_p_, and *M*_f_ stand for the nanoparticle and fluid densities and the nanoparticle and fluid molar masses, respectively. It is worthy to note that it is not necessary to know the molar mass and density of the nanoparticles to calculate the definitions in this Table and that it is mainly a question of size. Nevertheless, the values of *ρ*_p_ and *M*_p'_ (from which *M*_p_ is obtained) are still given in Table 1 should one need to know the values of ${\Gamma}_{{{{\mathrm{p}}}},{{{\mathrm{max}}}}}^{{{\mathrm{{\Sigma}}}}}$ and *Γ*_b_^Σ^ in terms of unit mass per unit surface. The values of *f*_∞_ have been adapted for the (dl)Au nanoparticles due to the presence of ligands at the gold surface inducing possible repulsion or blocking mechanisms. In ref. ^47^, it has been established that the (dl)Au nanoparticles occupy 0.2, 0.34, and 0.72 of the theoretical maximum coverage when dispersed in D, DD, and HD, respectively. Therefore, for the (dl)Au-D, (dl)Au-DD and (dl)Au-HD systems, *f*_∞_ has been multiplied by 0.2, 0.34, and 0.72, respectively. It is recalled that *δ*_m_ and *Φ*_m_ represent the primary minimum of the total potential energy and *Φ*_m_ its value, whereas *k*_B_ and *T* are Boltzmann’s constant and the temperature.

**Table 5:** Calculated parameters used in Eq. (25) for the nanoparticle dispersions.

NP-L	*γ*_f_ [mN m^−1^]	*K*_Σ_ [10^−3^]	*K*_p_ [10^2^]	*ω*_p_ [10^3^]	*Γ*_b_^Σ^ [nmol m^−2^]	*T* [K]
Al_2_O_3_-W	72.3	2.11	0.0562	6.23	148	300
Al_2_O_3_-D	23.8	4.66	11.5	1.28	67.1	300
Al-D	23.8	12.9	3.19	0.165	186	300
B-D	23.8	9.22	4.16	2.08	38.0	300
Al_2_O_3_-E	22.4	3.12	0.294	2.85	100	300
Al-E	22.4	8.66	0.103	0.369	279	300
B-E	22.4	6.17	0.0988	4.65	56.8	300
Al_2_O_3_-TEG	44.45	10.3	10.3	0.262	190	298
MgO-TEG	44.25	10.3	4.34	0.262	190	298
SiO_2_-W	72.5	3.51	O (0)	2.24	247	298
Ag-W	68.0	1.05	0.209	24.9	74.1	298
Lap-W	73.6	43.5	193	0.872	85.1	300
ZnO-EG	47.3	2.29	0.0964	5.27	75.9	300
(dl)Au-D	22.96	27.7	8726	0.036	399	303
(dl)Au-DD	24.75	29.2	8.55 * 10^4^	0.033	380	303
(dl)Au-HD	26.96	31.8	2.23*10^6^	0.027	349	303
Al_2_O_3_-Ws	72.3	2.64	O (0)	3.99	185	300

The symbols W, Ws, D, DD, HD, E, TEG, EG and G stand for the base fluids water, extra stabilised dispersion, n-decane, n-dodecane, n-hexadecane, ethanol, tri-ethylene glycol, ethylene glycol, and glycerol, respectively. Lap stands for laponite and (dl)Au for dodecanethiol-ligated gold. Note that *K*_p_ values that are orders of magnitude smaller than unity have been considered here as being virtually zero, *O*(0), i.e. negligibly small.

### Reporting summary

Further information on research design is available in the Nature Research Reporting Summary linked to this article.

## Results and discussion

### Comparison of model with experimental data

Gibbs adsorption isotherm ${{{\mathrm{d}}}}\gamma = - {\Gamma}_{{{\mathrm{p}}}}{{{\mathrm{d}}}}\eta _{{{\mathrm{p}}}}^{{{\mathrm{{\Sigma}}}}}$ can now be integrated. We use Eq. (6) for *Γ*_p_ (with (19) for *θ*_*p*_) and Eq. (12) for $\eta _{{{\mathrm{p}}}}^{{{\mathrm{{\Sigma}}}}}$. The surface tension of nanoparticle dispersions is finally given by

25 $$\gamma = \gamma _{{{\mathrm{f}}}} + R_{{{\mathrm{g}}}}T{\Gamma}_{{{\mathrm{b}}}}^{{{\mathrm{{\Sigma}}}}}\left( {\frac{{\omega _{{{\mathrm{p}}}} + \omega _{{{\mathrm{p}}}}K_{{{\mathrm{p}}}}\left( {\varphi + K_{{{\mathrm{{\Sigma}}}}}} \right) - K_{{{\mathrm{p}}}}K_{{{\mathrm{{\Sigma}}}}}}}{{1 + K_{{{\mathrm{p}}}}\varphi }}\varphi - \frac{{\omega _{{{\mathrm{p}}}} + \omega _{{{\mathrm{p}}}}K_{{{\mathrm{p}}}}K_{{{\mathrm{{\Sigma}}}}} - 1}}{{K_{{{\mathrm{p}}}}}}\ln \left( {1 + K_{{{\mathrm{p}}}}\varphi } \right)} \right)$$

where *γ*_f_ is the surface tension of the base fluid, *R*_g_ the universal gas constant, *T* the temperature, *K*_Σ_ given by (8), *K*_p_ given by (20), *ω*_p_ given by (13) and *φ* the nanoparticle volume fraction. The equilibrium constant is a kinetic parameter obtained by models from the literature, summarised in ‘*Surface kinetics*’. The other parameters are developed in this work using geometric principles and characteristic length scales, which would, for clarity, benefit from a summary in Table 4.

Table 5 shows the nanoparticle dispersions that we consider in this work. For completeness, the calculated numerical parameters that are necessary for determining the surface tension as a function of the volume fraction, i.e. *γ*_f_, *K*_Σ_, *ω*_p_ and ${\Gamma}_{{{\mathrm{b}}}}^{{{\mathrm{{\Sigma}}}}}$, are given in Table 5 for these nanoparticle dispersions.

**Fig. 3:**
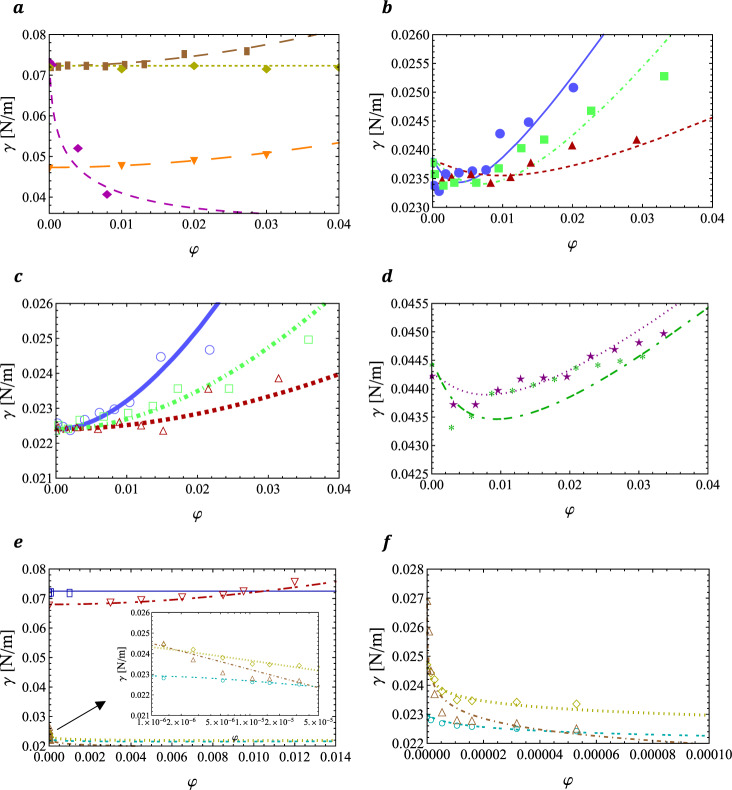
Modelled surface tension as a function of the volume fraction of nanoparticle dispersions, compared to experimental data^6,9–11,19,38,47,48^. **a** Al_2_O_3_-W (, ), Al_2_O_3_-Ws (, ), Laponite-W (, ) and ZnO-EG (,), **b** Al_2_O_3_-D (, ), Al-D (, ) and B-D (, ), **c** Al_2_O_3_-E (, ), Al-E (, ) and B-E (, ), **d** Al_2_O_3_-TEG (,*) and MgO-TEG (,), **e** SiO_2_-W (, ), Ag-W (, ), (dl)Au-D (, ), (dl)Au-DD (, ) and (dl)Au-HD (, ), the inset being a zoom of the (dl)Au-D system concentrating at the region of smaller *φ*-values, **f** a magnification of the systems (dl)Au-D (, ), (dl)Au-DD (, ) and (dl)Au-HD (, ). The studied systems are indicated in the form “nanoparticle-fluid”. The model values are indicated by lines, while the experimental data are given by markers in the form (line,marker).

Different kinds of behaviours for the surface tension of nanoparticle dispersions are represented by several experimental case studies, representing different materials (for both the nanoparticles and liquids) with different sizes^6,9–11,19,38,47,48^. The surface tension is calculated from Eq. (25) for these nanoparticle dispersions and compared to the experimental data in Fig. 3a–f. The experimental data in Fig. 3 show different types of behaviours and the present model has an overall good agreement with those data. This motivates to use the model to explain these observations.

### Non-occupancy contribution

We can divide the surface chemical potential, $\eta _{{{\mathrm{p}}}}^{{{\mathrm{{\Sigma}}}}} \equiv \eta _{{{\mathrm{p}}}} + \eta _{{{{\mathrm{NO}}}}}$, in a part that stands for the contribution by non-occupied sites *η*_NO_ ≡ −*ω*_p_*R*_g_*Tln*(1 − *θ*_p_) and a part that represents the contribution of the adsorbed nanoparticles *η*_p_ ≡ *R*_g_*Tln*(*θ*_p_). We can also split the surface tension change into two parts as *γ* − *γ*_f_ = ∆*γ*_p_ + ∆*γ*_NO_, where

26 $${\Delta}\gamma _{{{\mathrm{p}}}} = - {\int}_0^\varphi {{\Gamma}_{{{\mathrm{p}}}}\frac{{\partial \eta _{{{\mathrm{p}}}}}}{{\partial \varphi }}{{{\mathrm{d}}}}\varphi = {\int}_0^\varphi {\left( { - {\Gamma}_{{{\mathrm{b}}}}^{{{\mathrm{{\Sigma}}}}}\frac{{\partial \eta _{{{\mathrm{p}}}}}}{{\partial \varphi }}} \right)\left( {\frac{{{\Gamma}_{{{\mathrm{p}}}}}}{{{\Gamma}_{{{\mathrm{b}}}}^{{{\mathrm{{\Sigma}}}}}}}} \right){{{\mathrm{d}}}}\varphi } }$$

27 $${\Delta}\gamma _{{{{\mathrm{NO}}}}} = - {\int}_0^\varphi {{\Gamma}_{{{\mathrm{p}}}}\frac{{\partial \eta _{{{{\mathrm{NO}}}}}}}{{\partial \varphi }}{{{\mathrm{d}}}}\varphi = {\int}_0^\varphi {\left( { - {\Gamma}_{{{\mathrm{b}}}}^{{{\mathrm{{\Sigma}}}}}\frac{{\partial \eta _{{{{\mathrm{NO}}}}}}}{{\partial \varphi }}} \right)\left( {\frac{{{\Gamma}_{{{\mathrm{p}}}}}}{{{\Gamma}_{{{\mathrm{b}}}}^{{{\mathrm{{\Sigma}}}}}}}} \right){{{\mathrm{d}}}}\varphi } }$$

Equations (26) and (27) show the multiplication of two terms in the integral. The term $( { - {\Gamma}_{{{\mathrm{b}}}}^{{{\mathrm{{\Sigma}}}}}\frac{{\partial \eta _{{{{\mathrm{NO}}}}}}}{{\partial \varphi }}} )$ in Eq. (27) stands physically for the change in the surface chemical potential of non-occupied adsorption sites *per unit surface* upon a change in the nanoparticle bulk concentration. It is worthy to note that this emphasises the influence that non-adsorbed bulk nanoparticles have on the surface chemical potential of non-occupied sites, called here the *non-occupancy effect*. For later use, we assign for this term the following symbol

28 $${{{\mathcal{H}}}}_{{{{\mathrm{NO}}}}} = - {\Gamma}_{{{\mathrm{b}}}}^{{{\mathrm{{\Sigma}}}}}\frac{{\partial \eta _{{{{\mathrm{NO}}}}}}}{{\partial \varphi }}$$

A larger *absolute* value of ${{{\mathcal{H}}}}_{{{{\mathrm{NO}}}}}$ means a greater non-occupancy effect, i.e. one bulk nanoparticle will have more impact on the surface energy (and thus the surface chemical potential) of the non-occupied sites. Note that an equivalent analysis can be made for the adsorbed nanoparticles contribution (see Eq. (26)) through the term $( { - {\Gamma}_{{{\mathrm{b}}}}^{{{\mathrm{{\Sigma}}}}}\frac{{\partial \eta _{{{\mathrm{p}}}}}}{{\partial \varphi }}} ) = {{{\mathcal{H}}}}_{{{\mathrm{p}}}}$, called the *occupancy effect*. The term $( {\frac{{{\Gamma}_{{{\mathrm{p}}}}}}{{{\Gamma}_{{{\mathrm{b}}}}^{{{\mathrm{{\Sigma}}}}}}}} )$ stands for the excess surface concentration normalised by the surface-equivalent of the bulk concentration. We will assign the following symbol to it

29 $${{{\mathcal{D}}}}_{{{\mathrm{p}}}} = \frac{{{\Gamma}_{{{\mathrm{p}}}}}}{{{\Gamma}_{{{\mathrm{b}}}}^{{{\mathrm{{\Sigma}}}}}}}$$

A positive value of ${{{\mathcal{D}}}}_{{{\mathrm{p}}}}$ means a high degree of adsorption of nanoparticles (decreasing the surface energy), while a negative value indicates a preference of nanoparticles to remain dispersed in the bulk. In summary, the sign of ${{{\mathcal{D}}}}_{{{\mathrm{p}}}}$ indicates whether the surface tension will increase or decrease and the value of ${{{\mathcal{H}}}}_{{{{\mathrm{NO}}}}}$ with what amplitude. As both depend on *φ*, it is easy to understand that the magnitude and variation of the surface tension might be different as a function of *φ*, generating the different observed trends. More interestingly, Table 5 shows that the several nanoparticle dispersions considered here have quite different values for the nanoparticle equilibrium adsorption constant *K*_p_. This implies that this property plays an important role in determining the behaviour of the surface tension. Note that the parameters ${{{\mathcal{D}}}}_{{{\mathrm{p}}}}$ and ${{{\mathcal{H}}}}_{{{{\mathrm{NO}}}}}$ also depend on the surface coverage *θ*_p_, which is linked to *φ* through *K*_p_. This encourages to consider *φ* and the property *K*_p_ as suitable parameters for the present analysis.

We should define a certain reference system that represents a nanoparticle dispersion of which we can change freely the parameters *φ* and *K*_p_ and monitor their influence on the behaviour of ${{{\mathcal{H}}}}_{{{{\mathrm{NO}}}}}$ and ${{{\mathcal{D}}}}_{{{\mathrm{p}}}}$ and therefore on that of the surface tension. To perform numeric demonstrations, allowing the quantification of our analysis, we may choose data from any dispersion. Only because the B-D system is an example of an interesting decrease-increase behaviour, its data are used for the present demonstration. As the discussion should be followed in a general sense, and we only use the physical properties of this dispersion but changing freely *K*_p_, it is appropriate to name it differently: the reference system *R*1.

**Fig. 4:**
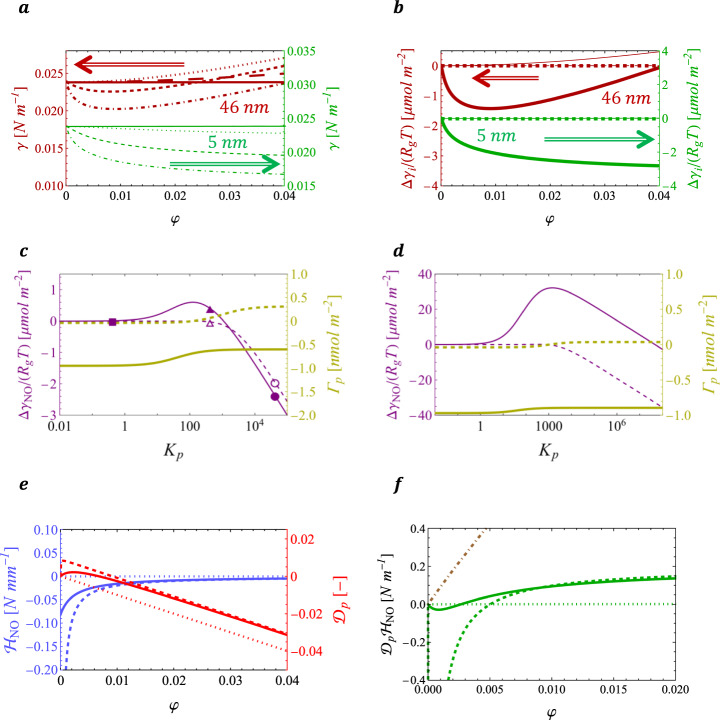
Analysis of surface tension behaviour. **a** *γ* of the *R*1 system vs *φ* for theoretically imposed *K*_p_ values ( =~0,  = 10,  = 10^2^,  = 10^3^,  = 5*10^3^ for 2*a*_p_ = 46 and 5 nm. **b** $\frac{{{\Delta}\gamma _{{{\mathrm{i}}}}}}{{R_{{{\mathrm{g}}}}T}}$ with *i* = *p,NO* vs *φ* of the *R*1 system, where thin and thick lines stand for *K*_p_ = 10 and 5 * 10^3^, respectively, and dashed and solid lines stand for *i* = *p* and *i* = *NO*, respectively, for 2*a*_p_ = 46 and 5 nm. Note that all the dashed lines are significantly horizontal. **c** $\frac{{{\Delta}\gamma _{{{{\mathrm{p}}}},{{{\mathrm{NO}}}}}}}{{R_{{{\mathrm{g}}}}T}}$ of the *R*1 system vs *K*_p_ for *φ* = 0.001 (dashed line) and *φ* = 0.025 (solid line) and 2*a*_p_ = 46 with three specific values of *K*_p_ based on the value from Table 5 (*K*_p*_ = 416): $\frac{{K_{{{{\mathrm{p}}}} \ast }}}{{1000}}$ ( for *φ* = 0.001 and  for *φ* = 0.025), *K*_p*_ ( for *φ* = 0.001 and  for *φ* = 0.025) and 100*K*_p*_ ( for *φ* = 0.001 and  for *φ* = 0.025). On the second axis, *Γ*_p_ of the reference system vs *K*_p_ for *φ* = 0.001 (dashed line) and *φ* = 0.025 (solid line) and 2*a*_p_ = 46, **d** $\frac{{{\Delta}\gamma _{{{{\mathrm{p}}}},{{{\mathrm{NO}}}}}}}{{R_{{{\mathrm{g}}}}T}}$ of the *R*2 system vs *K*_p_ for *φ* = 0.001 (dashed line) and *φ* = 0.025 (solid line) and 2*a*_p_ = 100. On the second axis, *Γ*_p_ of the *R*2 system vs *K*_p_ for *φ* = 0.001 (dashed line) and *φ* = 0.025 (solid line) and 2*a*_p_ = 100, **e** ${{{\mathcal{H}}}}_{{{{\mathrm{NO}}}}}$ vs *φ*, and ${{{\mathcal{D}}}}_{{{\mathrm{p}}}}$ vs *φ*, for three specific values of *K*_p_: $\frac{{K_{{{{\mathrm{p}}}} \ast }}}{{1000}}$ (dotted line), *K*_p*_ (solid line) and 100*K*_p*_ (dashed line) for the *R*1 system, **f** ${{{\mathcal{D}}}}_{{{\mathrm{p}}}}{{{\mathcal{H}}}}_{{{{\mathrm{NO}}}}}$ vs *φ* for three specific values of *K*_p_: $\frac{{K_{{{{\mathrm{p}}}} \ast }}}{{1000}}$ (dotted line), *K*_p*_ (solid line) and 100*K*_p*_ (dashed line) for the reference system and ${{{\mathcal{D}}}}_{{{\mathrm{p}}}}{{{\mathcal{H}}}}_{{{{\mathrm{NO}}}}}$ for *K*_p_ = *K*_p*_ (dotdashed line) for the Ag-W system.

Figure 4a shows the surface tension of the *R*1 system as a function of *φ* for various imposed *K*_p_ values and two different nanoparticle sizes.

Note that for small *K*_p_ values, the surface tension remains significantly constant. As this is counter intuitive (usually non-adsorption should lead to an increase in the surface tension as this entails that *Γ*_p_ < 0), special attention will be given to the small *K*_p_-case later. Figure 4a shows that, as *K*_p_ increases, *γ*(*φ*), for a given *φ*, first increases and then starts to decrease for small *φ*, followed by an overall decrease in the depicted *φ* -range. This first increase is also counter intuitive (usually more adsorption should lead to a decrease of the surface tension), a second point given special attention later. As *K*_p_ continues to increase, even a minimum in *γ*(*φ*) as a function of *φ* is observed, a third point discussed later as well. For even higher *K*_p_, the surface tension shows a decreasing trend, which is what one would expect. Figure 4a also shows that a smaller nanoparticle size tends to favour a decrease in the surface tension. The latter effect can be understood by noticing that smaller nanoparticles will increase, for the same *φ*, the number of nanoparticles and therefore also the number of adsorbed nanoparticles, which leads eventually (for sufficiently small nanoparticles) to a decrease in the surface tension.

Let us, before entering into such an analysis, first determine what contribution to the surface tension is more important, ∆*γ*_p_ or ∆*γ*_NO_. Figure 4b shows, through ∆*γ*_i_ (*i* = *p*,*NO*) scaled by *R*_g_*T*, that the contribution of the non-occupied sites (*i* = *NO*, the solid lines) is the main one, especially at larger *K*_p_-values. The main reason for this is size-related. The nanoparticles are much larger than the fluid molecules, which constitute the adsorption sites. This means that the number of fluid molecules involved in the adsorption of a nanoparticle is quite large, expressed in large *ω*_p_-values, i.e. *ω*_p_ ≫ 1, as Table 5 shows. So, it is now evident that the non-occupied site contribution of the surface chemical potential will be a key part in the following discussions.

### Three counter-intuitive effects of *K*_p_ on surface tension

Figure 4c shows ∆*γ*_NO_ and *Γ*_p_ for two volume fractions for the *R*1 system as a function of *K*_p_. To facilitate the discussion three markers have been introduced for ∆*γ*_NO_: one corresponding to the *K*_p_ from Table 5 for the B-D system (≡*K*_p*_), a smaller $\frac{{K_{{{{\mathrm{p}}}} \ast }}}{{1000}}$ one and a larger 100*K*_p*_ one. Figure 4d represents ∆*γ*_NO_ and *Γ*_p_ for two volume fractions as a function of *K*_p_ for a so-called *R*2 system, where we use the data from the Ag-W system, merely to illustrate that *K*_p_ has the same type of effect on the surface tension for whatever nanoparticle dispersion’s physical properties. Figure 4e shows ${{{\mathcal{H}}}}_{{{{\mathrm{NO}}}}}$, ${{{\mathcal{D}}}}_{{{\mathrm{p}}}}$ and ${{{\mathcal{D}}}}_{{{\mathrm{p}}}}{{{\mathcal{H}}}}_{{{{\mathrm{NO}}}}}$ (combined contribution of the latter two) versus *φ* for three *K*_p_ values for the *R*1 system.

For small $K_{{{\mathrm{p}}}}( { = \frac{{K_{{{{\mathrm{p}}}} \ast }}}{{1000}}} )$, Fig. 4e shows that ${{{\mathcal{D}}}}_{{{\mathrm{p}}}}$ is significantly negative over the whole volume fraction range. Figure 4e shows that at small *K*_p_ there is a negligible contribution of the absolute value (being, by the way, always negative) of ${{{\mathcal{H}}}}_{{{{\mathrm{NO}}}}}$ (dotted blue line), it is significantly constant over the *φ* range. Although ${{{\mathcal{D}}}}_{{{\mathrm{p}}}}$ is clearly negative (dotted red line in Fig. 4e), which stands for a negative surface excess concentration and would conventionally imply an increase in the surface tension, the resulting surface tension remains significantly constant as shown by Fig. 4a (straight solid lines). To understand why this is, we take the limit of Eq. (25) for *K*_p_ → 0, which gives

30 $$\gamma _{K_{{{\mathrm{p}}}} \to 0} = \mathop {{\lim }}\limits_{K_{{{\mathrm{p}}}} \to 0} \gamma = \gamma _{{{\mathrm{f}}}} + R_{{{\mathrm{g}}}}T{{{\mathrm{{\Gamma}}}}}_{{{\mathrm{b}}}}^{{{\mathrm{{\Sigma}}}}}\varphi$$

Filling in Eq. (30) the data for the *R*1 system reveals that $\gamma _{K_{{{\mathrm{p}}}} \to 0} - \gamma _{{{\mathrm{f}}}} = O(10^{ - 5} - 10^{ - 4})\varphi$. This explains the seemingly (in reality, very weakly increasing) constant value of the surface tension. The reason behind the seemingly constant value of the surface tension at *K*_p_ → 0 lies in the value of *Γ*_b_^Σ^. From Table 4 one may easily deduce that ${\Gamma}_{{{\mathrm{b}}}}^{{{\mathrm{{\Sigma}}}}} \propto \frac{1}{{a_{{{\mathrm{p}}}}\ell _{{{\mathrm{f}}}}}}$. Nanoparticles have generally a larger size than fluid molecules and apparently large enough for *Γ*_b_^Σ^ to be sufficiently small and hence a seemingly constant behaviour of the surface tension can be predicted. *This explains the first counter-intuitive observation*.

At *K*_p_ = *K*_p*_, Fig. 4e shows that for a small range of *φ* we have ${{{\mathcal{D}}}}_{{{\mathrm{p}}}}\, > \,0$ (equivalent to *Γ*_p_ > 0, let us recall), while $\left| {{{{\mathcal{H}}}}_{{{{\mathrm{NO}}}}}} \right|$ becomes bigger than for the previous case (see solid line in Fig. 4e as opposed to the dotted line). As ${{{\mathcal{H}}}}_{{{{\mathrm{NO}}}}} < 0$ (always), this leads to ${{{\mathcal{D}}}}_{{{\mathrm{p}}}}{{{\mathcal{H}}}}_{{{{\mathrm{NO}}}}}\, < \,0$ (solid line in Fig. 4f). As we increase *φ*, Fig. 4e shows that ${{{\mathcal{D}}}}_{{{\mathrm{p}}}}$ changes sign, i.e. ${{{\mathcal{D}}}}_{{{\mathrm{p}}}}\, < \,0$, with $\left| {{{{\mathcal{H}}}}_{{{{\mathrm{NO}}}}}} \right|$ still being significantly larger than zero, resulting into ${{{\mathcal{D}}}}_{{{\mathrm{p}}}}{{{\mathcal{H}}}}_{{{{\mathrm{NO}}}}}\, > \,0$. As the surface tension depends on the integration of ${{{\mathcal{D}}}}_{{{\mathrm{p}}}}{{{\mathcal{H}}}}_{{{{\mathrm{NO}}}}}$ from 0 to *φ*, the surface tension will decrease as long as ${{{\mathcal{D}}}}_{{{\mathrm{p}}}}{{{\mathcal{H}}}}_{{{{\mathrm{NO}}}}}\, < \,0$ and increases as long as ${{{\mathcal{D}}}}_{{{\mathrm{p}}}}{{{\mathcal{H}}}}_{{{{\mathrm{NO}}}}}\, > \,0$, passing thus through a minimum. This analysis implies that the sign of the integrated surface of ${{{\mathcal{D}}}}_{{{\mathrm{p}}}}{{{\mathcal{H}}}}_{{{{\mathrm{NO}}}}}$ as a function of *φ* will determine the existence and positioning of a surface tension minimum. It is then logical to elaborate further on the dependence of ${{{\mathcal{H}}}}_{{{{\mathrm{NO}}}}}$ and ${{{\mathcal{D}}}}_{{{\mathrm{p}}}}$ on *φ*. From Eqs. (6) and (19), we have that

31 $${{{\mathcal{D}}}}_{{{\mathrm{p}}}} = \frac{{K_{{{\mathrm{p}}}}\varphi }}{{1 + K_{{{\mathrm{p}}}}\varphi }}K_{{{\mathrm{{\Sigma}}}}} - \varphi$$

and from Eqs. (19) and (28) that

32 $${{{\mathcal{H}}}}_{{{{\mathrm{NO}}}}} \propto - {\Gamma}_{{{\mathrm{b}}}}^{{{\mathrm{{\Sigma}}}}}\frac{{K_{{{\mathrm{p}}}}\omega _{{{\mathrm{p}}}}}}{{1 + K_{{{\mathrm{p}}}}\varphi }}$$

The analysis of Eqs. (31) and (32) needs some mathematical considerations. From Eq. (31), we can easily deduce that ${{{\mathcal{D}}}}_{{{\mathrm{p}}}} = 0$ when $\varphi = \frac{{K_{{{\mathrm{p}}}}K_{{{\mathrm{{\Sigma}}}}} - 1}}{{K_{{{\mathrm{p}}}}}}$ or *φ* = 0, but the latter is a trivial solution not considered further. The sign of ${{{\mathcal{D}}}}_{{{\mathrm{p}}}}$ depends on the values of *K*_p_ and *K*_Σ_. With respect to this, two cases can be considered: $K_{{{\mathrm{p}}}} \le \frac{1}{{K_{{{\mathrm{{\Sigma}}}}}}}$ and $K_{{{\mathrm{p}}}} > \frac{1}{{K_{{{\mathrm{{\Sigma}}}}}}}$. These cases will depend on the parameter *K*_Σ_. From Table 4, we can deduce that $K_{{{\mathrm{{\Sigma}}}}} \propto f_\infty \frac{{\ell _{{{\mathrm{f}}}}}}{{a_{{{\mathrm{p}}}}}}$. The values of parameter *f*_∞_ (see Table 4) are constant for a certain shape and *K*_Σ_ will depend on the ratio $\frac{{\ell _{{{\mathrm{f}}}}}}{{a_{{{\mathrm{p}}}}}}$ much more than on *f*_∞_. Therefore, for the discussion of Eq. (31) we will only take into consideration $K_{{{\mathrm{{\Sigma}}}}} \propto \frac{{\ell _{{{\mathrm{f}}}}}}{{a_{{{\mathrm{p}}}}}}$

We treat the case $K_{{{\mathrm{p}}}} \le \frac{1}{{K_{{{\mathrm{{\Sigma}}}}}}}$. When $K_{{{\mathrm{p}}}} \le \frac{1}{{K_{{{\mathrm{{\Sigma}}}}}}}$, we have $\frac{{K_{{{\mathrm{p}}}}K_{{{\mathrm{{\Sigma}}}}} - 1}}{{K_{{{\mathrm{p}}}}}} \le 0$ and it can be verified that this means that for all *φ* > 0 we have ${{{\mathcal{D}}}}_{{{\mathrm{p}}}}\, < \,0$. As ${{{\mathcal{H}}}}_{{{{\mathrm{NO}}}}}$ is always negative, the result is a strictly increasing surface tension. Depending on the amplitude of $\left| {{{{\mathcal{H}}}}_{{{{\mathrm{NO}}}}}} \right|$, this increase will be significantly measurable or not. Focussing mainly on the value of *K*_p_ (the value of which may vary orders of magnitude more than *ω*_p_ and *Γ*_b_^Σ^, see Table 5), two cases are thus possible: vanishing *K*_p_ values (*K*_p_ → 0) and non-vanishing *K*_p_ values ($O(0) \ll K_{{{\mathrm{p}}}} \le \frac{1}{{K_{{{\mathrm{{\Sigma}}}}}}}$, where *O*(0) stands for a value that is so low that considering it zero would reflect a measured reality).

$${\mathrm{o}}\quad K_{{{\mathrm{p}}}} \to 0$$

Equation (32) shows that for small *K*_p_ (e.g. *K*_p_ → 0), we have $\left| {{{{\mathcal{H}}}}_{{{{\mathrm{NO}}}}}} \right| \to 0$, so that the surface tension increase is not noticeable. This has been discussed previously around Eq. (30) for small *K*_p_ values and a real example for this is the SiO_2_-W system (see Fig. 3e Table 5 where indeed $K_{{{\mathrm{p}}}} \ll \frac{1}{{K_{{{\mathrm{{\Sigma}}}}}}}$).

$${\mathrm{o}}\quad {O}(0) \ll K_{{{\mathrm{p}}}} \le \frac{1}{{K_{{{\mathrm{{\Sigma}}}}}}}$$

When *K*_p_ is significantly non-zero but not too large, i.e. $O(0) \ll K_{{{\mathrm{p}}}} \le \frac{1}{{K_{{{\mathrm{{\Sigma}}}}}}}$ (defined as the lower-intermediary region), so that it can be verified that $\left| {{{{\mathcal{H}}}}_{{{{\mathrm{NO}}}}}} \right|$ has a significant value, the increase of the surface tension will be measurable. A real example for this is the Ag-W system, where Table 5 shows that *K*_p_ ≫ *O*(0) and $K_{{{\mathrm{p}}}} < \frac{1}{{K_{{{\mathrm{{\Sigma}}}}}}}$. Moreover, Fig. 4f illustrates this as well by a continuously increasing ${{{\mathcal{D}}}}_{{{\mathrm{p}}}}{{{\mathcal{H}}}}_{{{{\mathrm{NO}}}}}$ (brown dot-dashed line). Departing from a fully desorbed case (*K*_p_ → 0), we can say that upon enhancement (*K*_p_ ≫ *O*(0)) of the surface adsorption kinetics (up to the limit $K_{{{\mathrm{p}}}} \le \frac{1}{{K_{{{\mathrm{{\Sigma}}}}}}}$), the surface tension behaviour becomes a strictly increasing one due to the combination of ${{{\mathcal{D}}}}_{{{\mathrm{p}}}} < 0$ and a significant value of $\left| {{{{\mathcal{H}}}}_{{{{\mathrm{NO}}}}}} \right|$. So, initially, a higher adsorption appears not to lead to a lower but rather to a higher surface tension. As $\frac{1}{{K_{{{\mathrm{{\Sigma}}}}}}} \propto \frac{{a_{{{\mathrm{p}}}}}}{{\ell _{{{\mathrm{f}}}}}}$, nanoparticles (having much higher size than the fluid molecules) allow for a much larger limit for *K*_p_ for which ${{{\mathcal{D}}}}_{{{\mathrm{p}}}}$ remains negative. So, within this limit, upon increasing *K*_p_, the strength of the non-occupancy effect $\left| {{{{\mathcal{H}}}}_{{{{\mathrm{NO}}}}}} \right|$ becomes significant, whilst the excess surface concentration remains ${{{\mathcal{D}}}}_{{{\mathrm{p}}}} < 0$, resulting into a surface tension increase and not decrease. *This explains the second counter-intuitive observation*.

We treat the case $K_{{{\mathrm{p}}}} > \frac{1}{{K_{{{\mathrm{{\Sigma}}}}}}}$. When $K_{{{\mathrm{p}}}} > \frac{1}{{K_{{{\mathrm{{\Sigma}}}}}}}$, we have a particular situation where ${{{\mathcal{D}}}}_{{{\mathrm{p}}}} > 0$ (and therefore a decreasing surface tension) for $0\, < \,\varphi\, < \,\frac{{K_{{{\mathrm{p}}}}K_{\Sigma} - 1}}{{K_{{{\mathrm{p}}}}}}$ and ${{{\mathcal{D}}}}_{{{\mathrm{p}}}}\, < \,0$ (increasing surface tension) for $\varphi\, > \,\frac{{K_{{{\mathrm{p}}}}K_{\Sigma} - 1}}{{K_{{{\mathrm{p}}}}}}$, where we limit *φ* to a certain maximum value *φ*_max_, considered reasonable for typical nanoparticle dispersions (as discussed later). It can be verified that the surface tension is strictly decreasing if $\frac{{K_{{{\mathrm{p}}}}K_{\Sigma} - 1}}{{K_{{{\mathrm{p}}}}}} \ge 1$, which means for $K_{\Sigma} \ge 1 + \frac{1}{{K_{{{\mathrm{p}}}}}}$. This gives two regions, the one given by the latter condition and $K_{\Sigma} < 1 + \frac{1}{{K_{{{\mathrm{p}}}}}}$.

$${\mathrm{o}}\quad K_{\Sigma}\, <\, 1 + \frac{1}{{K_{{{\mathrm{p}}}}}}$$

The values given in Table 5 show that for nanoparticle dispersions typically *K*_Σ_ ≪ 1. This means that *K*_p_ ≫ 1. The smaller $\frac{{K_{{{\mathrm{p}}}}K_{\Sigma} - 1}}{{K_{{{\mathrm{p}}}}}}$ (due to larger nanoparticles as $K_{\Sigma} \propto \frac{{\ell _{{{\mathrm{f}}}}}}{{a_{{{\mathrm{p}}}}}}$ or due to lower *K*_p_) is, the closer the value of *φ* for which ${{{\mathcal{D}}}}_{{{\mathrm{p}}}}$ changes sign will be to zero. As a consequence, this fits within the typical operating *φ*-ranges (*φ* < *φ*_max_), resulting into an observable minimum for the surface tension. This is the case for e.g. the B-D, Al-D, Al_2_O_3_-D systems (see Fig. 3b and Table 5 for the values). As, however, $\frac{{K_{{{\mathrm{p}}}}K_{\Sigma} - 1}}{{K_{{{\mathrm{p}}}}}}$ becomes somewhat larger (smaller nanoparticles or higher *K*_p_) the *φ* for which ${{{\mathcal{D}}}}_{{{\mathrm{p}}}}$ changes sign will increase and may fall out of the aforementioned typical operating *φ*-ranges (*φ* > *φ*_max_). This results into a minimum that is no longer observed (mathematically still present, but experimentally not observed within typical *φ*-ranges) and the surface tension is *virtually* decreasing. This can also be numerically verified in Table 5 and visually in Fig. 3a for e.g. the Lap-W system. For systems with even higher *K*_p_, Eq. (32) shows that $\left| {{{{\mathcal{H}}}}_{{{{\mathrm{NO}}}}}} \right|$ as well as the negative part of ${{{\mathcal{D}}}}_{{{\mathrm{p}}}}{{{\mathcal{H}}}}_{{{{\mathrm{NO}}}}}$ become more important, confirmed by the 100*K*_p*_ case for the *R*1 system in Fig. 4(e) and (f) (dashed lines). The (dl)Au dispersions (see again Table 5 and also Fig. 3(e) and (f)) illustrate this situation by presenting surface tensions that decrease quickly for very low volume fractions. In summary, this means that an observable surface tension minimum is the result of a delicate balance between a sufficiently large, but not too small, nanoparticle (through $K_{\Sigma} \propto \frac{{\ell _{{{\mathrm{f}}}}}}{{a_{{{\mathrm{p}}}}}}$) and a sufficient amount of adsorption (through $K_{{{\mathrm{p}}}} > \frac{1}{{K_{\Sigma}}}$). This effect is therefore not an external one but stems from the same parameters that cause strictly increasing or decreasing behaviours, merely because the conditions are right. *This explains the third counter-intuitive observation*.

$${\mathrm{o}}\quad K_{\Sigma} \ge 1 + \frac{1}{{K_{{{\mathrm{p}}}}}}$$

Mathematically speaking, a strict decrease (over the whole range 0 < *φ* < 1) in the surface tension would occur if, next to $K_{{{\mathrm{p}}}} > \frac{1}{{K_{\Sigma}}}$, we have $K_{\Sigma} \ge 1 + \frac{1}{{K_{{{\mathrm{p}}}}}}$. We have mentioned earlier that typically *K*_p_ ≫ 1. This means that, as approximation, we are practically dealing here with the condition *K*_Σ_ ≥ 1, which entails that $a_{{{\mathrm{p}}}} \le O(\ell _{{{\mathrm{f}}}})$. This would besides possibly quantum dots or surfactants, be rather untypical for nanoparticle dispersions. Therefore, this case can be disregarded as well for nanoparticle dispersions in general.

### Trends and comments

In fine, it seems that the right combination between adsorption strength (*K*_p_) and nanoparticle size (*a*_p_ of which the main effect is represented by the parameter *K*_Σ_) is responsible for the different behaviours. Table 6 shows a summary of the different surface tension behaviours as a function of the parameters *K*_p_ and *K*_Σ_, in the form of the product *K*_p_*K*_Σ_.

**Table 6:**
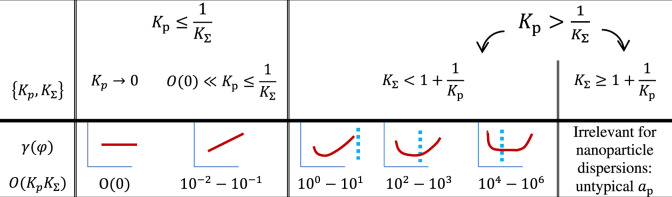
Surface tension behaviours as function of *K*_p_ (adsorption) and *K*_Σ_ (size).

Table 6 shows that as one goes from left to right, the value of *K*_p_*K*_Σ_ increases by several orders of magnitude. This is by either increasing *K*_p_, *K*_Σ_ or both. Table 5 shows that *K*_Σ_ for all the nanoparticle dispersions is of the order of 10^−2^−10^−3^. This means that when *K*_p_*K*_Σ_ increases several orders of magnitude, this is mainly due to *K*_p_. Nevertheless, for quantitative assessments, it is more convenient to mention *K*_p_*K*_Σ_.

In order to put the results in perspective, some additional comments are in place here. In the case $K_{{{\mathrm{p}}}}\, > \,\frac{1}{{K_{{{\mathrm{{\Sigma}}}}}}}$ and $K_{\Sigma}\, < \,1 + \frac{1}{{K_{{{\mathrm{p}}}}}}$, we have made a distinction for the surface tension behaviour between three ranges of orders of magnitude for *K*_p_*K*_Σ_. *Mathematically*, these three cases represent all a minimum in the surface tension somewhere in the range 0 < *φ* < 1. The reason for making the three distinctions is on a conceptual level, involving measured data and a defined framework. As Fig. 3 shows, most of nanoparticle dispersions that are used for engineering purposes (one might also think of medical ones as well, for that matter) present *operating conditions* that involve *φ* values that are often limited by a value *φ*_max_ that is of the order of *φ*_max_ ≈ O(10^−2^) or slightly higher, but still *φ*_max_ < O(10^−1^). In Table 6, *φ*_max_ is schematically indicated for the case $K_{{{\mathrm{p}}}}\, > \,\frac{1}{{K_{\Sigma}}}$ and $K_{\Sigma} < 1 + \frac{1}{{K_{{{\mathrm{p}}}}}}$ by blue vertical dotted lines, set to a same hypothetical value for the three images in question. It shows that as *K*_p_*K*_Σ_ increases the minimum of the surface tension becomes less pronounced and shifts towards higher *φ* values (not on scale), falling out of the range limited by *φ*_max_. At *φ* values beyond *φ*_max_ it is the question whether we can still speak of dispersions and we then might have to deal with another type of “fluid” with additional phenomena at the surface. When working with nanoparticle dispersions, we have limited the analysis within the range 0 < *φ* < *φ*_max_ (named the “operating range”). As such, depending on the value of *K*_p_*K*_Σ_, the mathematical minimum of the surface tension may well be out of that range and therefore not observed nor experimentally measured. Then, it is justified to indicate conditions (that is, within the range 0 < *φ* < *φ*_max_), where we can observe a minimum in the surface tension (for *K*_p_*K*_Σ_ ≈ *O*(10^0^−10^1^)) and where we observe a virtual decrease. Even for the virtual decrease of the surface tension, we have made a distinction between a “soft” decrease (*K*_p_*K*_Σ_ ≈ *O*(10^2^−10^3^)) and a “steep” decrease (*K*_p_*K*_Σ_ ≈ *O*(10^4^−10^6^), the upper limit 10^6^ being indicative with respect to the observed experiments, but may conceptually be even higher). The soft decrease is defined as the surface tension having a steady decrease over the whole operating range, such as the Lap-W case. The steep decrease is characterised by a strong decrease of the surface tension for *φ* ≪ *O*(*φ*_max_) with a seemingly constant value afterwards, such as the dl(Au) dispersions.

For the parameter *K*_Σ_, we have mentioned that for nanoparticles we have $K_{\Sigma} \propto \frac{{\ell _{{{\mathrm{f}}}}}}{{a_{{{\mathrm{p}}}}}}$, not considering *f*_∞_ in the discussions. There are, however, cases where this parameter may play a role. When strong repulsive forces are present or when the nanoparticle surfaces (because of their nature or their functionalization) are such that we cannot consider them as hard spheres, the maximum coverage may, respectively, decrease or increase, whereas the shape may also be altered by the stretching or compressing of the adsorbed nanoparticles. In such cases, additional considerations should be made in order to include these forces between nanoparticles^49^. One may say that these forces will be effective there where the nanoparticles are present at the interface, so that the surface tension of particle-laden interfaces is argued to be an effective magnitude^49^. In some cases, the nanoparticles are grafted with polymers, which may cause additional effects on the surface tension due to the dangling chains of the polymers^50^. When ions are present (one may think of electrolytes or charged organic molecules) strong coulomb interactions may also influence the maximum coverage.

The effect of the nanoparticle size has been mainly expressed through the parameter *K*_Σ_. It should be recalled that the nanoparticle size also figures in the parameter *ω*_p_. The parameter *ω*_p_ (standing for the number of adsorption sites) can also quantitatively interfere with the magnitude of the surface tension change through its linear relation with the non-occupancy effect Eq. (32). Moreover, the parameter *ω*_p_, when much larger than unity, is responsible for the non-occupancy effect to outnumber the occupancy effect through the surface chemical potential (see Eq. (12)). Should it be around unity, the free energy contribution of the adsorbed nanoparticles would also be important for the chemical potential and our discussion would be different. Nevertheless, once it is established that for nanoparticles generally *ω*_p_ ≫ 1, and that the variation of *K*_p_ is, as mentioned earlier, of far more importance for the non-occupancy effect, the variation of the parameter *ω*_p_ is not given more attention in our analysis.

The size of the nanoparticles also matters from another point of view. The projection method necessitates that the radius of curvature (reciprocal of the curvature) should be much larger than the nanoparticle radius. In other words, the interface should be “flat” with respect to the size of the nanoparticles. If the pressure difference over the interface is negligible, Young’s equation (where the pressure difference is related to the surface tension and the interface curvature) predicts that such an assumption would be realistic.

In ‘Representation of an interfacial layer on a dividing surface’, we mentioned that we used half the surface to calculate the volume-to-surface ratio. A heuristic reason was employed for this, assuming that only half the surface facing the dividing surface would matter in the adsorption process for particles that are much larger than the fluid molecules that constitute the adsorption sites. As a verification, we performed surface tension calculations using cases with a fourth, a sixth and the whole particle’s surface to calculate the volume-to-surface ratio. It appeared that the heuristic choice we have made for the calculation of the volume-to-surface ratio, i.e. using half the nanoparticle’s surface, was the most appropriate one with respect to the experimental data. It would be interesting to investigate the degree of this participating surface experimentally. However, for this work, the heuristic choice we have made appeared to be sufficient.

It should be noted that the way *K*_p_ has been calculated assumes that it is enough to take into account the wettability of the nanoparticles in the potential energy. The DLVO theory is known to be used for adsorption on solid-liquid interfaces. In refs. ^24,51^ as well as in the present work, it is assumed that the DLVO theory, albeit extended, is applicable for liquid-fluid interfaces as well. Although already used by others^24,51^, such a kinetic model should be studied in more details. In addition, it would be encouraged to provide benchmark studies with experimental data on the adsorption coefficients of various nanoparticle adsorption on liquid–air (fluid) interfaces.

Finally, the present model considers the adsorption of nanoparticles alone in order to focus on this phenomenon. It would be interesting to generalise or adapt Eq. (3) for the inclusion of the adsorption of molecular species, which could generalise the model for the application of studying the surface tension of solutions containing surfactants or other (in)organic molecules. Should multiple adsorption occur, the thermodynamic model presented in this work lends itself to be extended starting, most importantly, from an adaptation of Eq. (3).

### Mapping of the surface tension behaviour

**Fig. 5:**
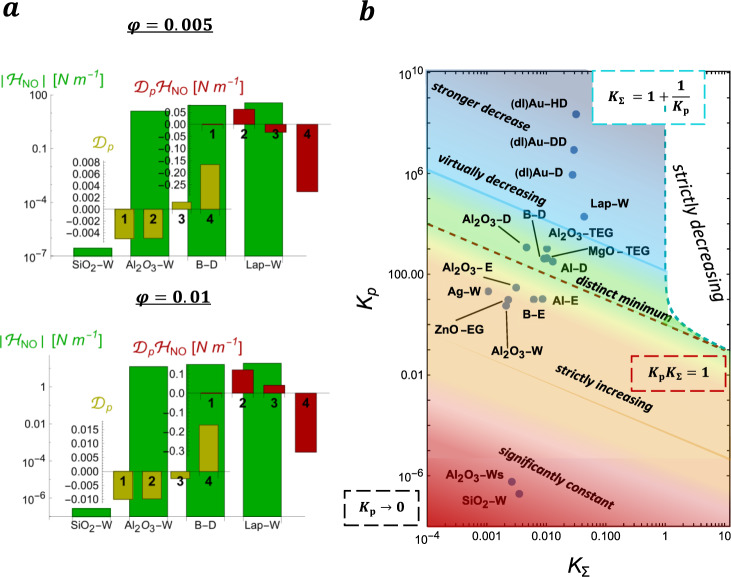
Mapping of surface tension behaviour. **a** Competition of key parameters $\left| {{{{\mathcal{H}}}}_{{{{\mathrm{NO}}}}}} \right|$, ${{{\mathcal{D}}}}_{{{\mathrm{p}}}}$ and ${{{\mathcal{D}}}}_{{{\mathrm{p}}}}{{{\mathcal{H}}}}_{{{{\mathrm{NO}}}}}$ for the nanoparticle dispersions SiO_2_-W (**1**), Al_2_O_3_-W (**2**), B-D (**3**) and Lap-W (**4**) for two volume fractions: *φ* = 0.005, *φ* = 0.01. Note that for a better visualisation both the values of ${{{\mathcal{D}}}}_{{{\mathrm{p}}}}$ and ${{{\mathcal{D}}}}_{{{\mathrm{p}}}}{{{\mathcal{H}}}}_{{{{\mathrm{NO}}}}}$ for only the Lap-W system have been divided by 5 and 2 for the cases *φ* = 0.005, *φ* = 0.01, respectively. **b** All the considered nanoparticle dispersion systems are resumed in a *K*_p_ − *K*_Σ_ map, representing five observed *γ*-vs-*φ* behaviours: “significantly constant” where the change (proven to be mathematically an increase) in *γ* is not observable (less than 1%) on the 1 mN/m range, “strictly increasing” where ∂_*φ*_*γ* > 0 over any *φ*-range, “distinct minimum” where a clear minimum is visible at operating *φ* ranges, “virtually decreasing” where ∂_*φ*_*γ* < 0 at operating *φ* ranges and “stronger decrease” where ∂_*φ*_*γ* decreases distinctly steeper than the previous case. It is to be reminded that the latter three cases are distinguished within the *φ*-range under which typical nanoparticle dispersions are used. Moreover, the latter two cases are conceptually the same but are distinguished for application or engineering purposes: much higher adsorption kinetics and/or smaller nanoparticles induce (although theoretically having the same tendency) for the observer a decrease that is much steeper and occurs at much lower nanoparticle concentrations, which justifies to make a distinction between them. The colours indicate qualitatively the transition from one region to another, whereas the model gives two mathematical limits: the limit *K*_p_*K*_Σ_ = 1 (red dashed line) designates formally the crossover from ∂_*φ*_*γ*|_∀*φ*_ > 0 to ∂_*φ*_*γ*|_*φ*→0_ < 0, while the limit $K_{\Sigma} = 1 + \frac{1}{{K_{{{\mathrm{p}}}}}}$ (blue dashed line) stands for the crossover from ∂_*φ*_*γ*|_*φ*→0_ < 0 to ∂_*φ*_*γ*|_∀*φ*_ < 0. Interestingly, **b** shows that the dispersions seem to correspond to sets of simultaneously increasing *K*_p_ and *K*_Σ_, indicated by the left-to-right diagonally up-going set of points. This confirms the link between *K*_p_*K*_Σ_ and the behaviour of the surface tension.

The previous analysis has shown a general dependence of the surface tension behaviour on *K*_p_*K*_Σ_, where *K*_Σ_ and *K*_p_ stand for the effect of nanoparticle size and adsorption strength, respectively. In order to quantify this dependence and map these behaviours, we choose four representative systems, having, respectively, seemingly constant, strictly increasing, minimum containing and virtually decreasing behaviours for the surface tension. Figure 5a shows the values of ${{{\mathcal{D}}}}_{{{\mathrm{p}}}}$, $\left| {{{{\mathcal{H}}}}_{{{{\mathrm{NO}}}}}} \right|$ and ${{{\mathcal{D}}}}_{{{\mathrm{p}}}}{{{\mathcal{H}}}}_{{{{\mathrm{NO}}}}}$ for these four nanoparticle dispersions, at two volume fractions, that have distinct behaviours with low to high *K*_p_*K*_Σ_ in the following order: SiO_2_-W < Al_2_O_3_-W < B-D < Lap-W. Figure 5a shows that, although SiO_2_-W and Al_2_O_3_-W have comparable negative ${{{\mathcal{D}}}}_{{{\mathrm{p}}}}$ values, ${{{\mathcal{D}}}}_{{{\mathrm{p}}}}{{{\mathcal{H}}}}_{{{{\mathrm{NO}}}}}$ is only significant for Al_2_O_3_-W due to a much higher $\left| {{{{\mathcal{H}}}}_{{{{\mathrm{NO}}}}}} \right|$, confirming the analysis in the previous section, which means a non-measurable increasing surface tension for SiO_2_-W and a measurable one for Al_2_O_3_-W. As *K*_p_*K*_Σ_ increases, i.e. for B-D, we can see a positive ${{{\mathcal{D}}}}_{{{\mathrm{p}}}}$ for *φ* = 0.005 and a negative one for *φ* = 0.01. As the value $\left| {{{{\mathcal{H}}}}_{{{{\mathrm{NO}}}}}} \right|$ is significant enough, this results into a visibly negative ${{{\mathcal{D}}}}_{{{\mathrm{p}}}}{{{\mathcal{H}}}}_{{{{\mathrm{NO}}}}}$ for *φ* = 0.005 and a positive one for *φ* = 0.01, meaning first a decrease and then an increase in the surface tension. For even larger *K*_p_*K*_Σ_, i.e. for Lap-W, we can see a positive ${{{\mathcal{D}}}}_{{{\mathrm{p}}}}$ for both *φ*’s. With a large $\left| {{{{\mathcal{H}}}}_{{{{\mathrm{NO}}}}}} \right|$, ${{{\mathcal{D}}}}_{{{\mathrm{p}}}}{{{\mathcal{H}}}}_{{{{\mathrm{NO}}}}}$ is considerably negative for both *φ*’s, corresponding to a virtually decreasing surface tension behaviour that was observed for Lap-W.

In the present study, we aimed at proposing a framework, model and explanation dealing with the different behaviours of the surface tension of nanoparticle dispersions. We have seen that the adsorption strength (*K*_p_*K*_Σ_) and the nanoparticle size (through *K*_Σ_) collaborate or compete in determining these different tendencies. It is then interesting to map the surface tension behaviours of all the nanoparticle dispersions that were presented in Fig. 3 as a function of *K*_p_ and *K*_Σ_. Such a mapping is presented in Fig. 5b and gives the opportunity to tailor nanoparticle dispersions, e.g. through size (affecting *K*_Σ_) and surface properties (affecting *K*_p_, since the surface of the nanoparticles have a direct influence on their adsorption strengths), for the envisioned effect of the surface tension.

## Supplementary information

Reporting Summary

## Data Availability

The data in this study are available upon reasonable request.

## Code availability

The commercial software Mathematica has been used to analyse the results.
